# Protein kinase R dependent phosphorylation of α-synuclein regulates its membrane binding and aggregation

**DOI:** 10.1093/pnasnexus/pgac259

**Published:** 2022-11-16

**Authors:** Lasse Reimer, Hjalte Gram, Nanna Møller Jensen, Cristine Betzer, Li Yang, Lorrain Jin, Min Shi, Driss Boudeffa, Giuliana Fusco, Alfonso De Simone, Deniz Kirik, Hilal A Lashuel, Jing Zhang, Poul Henning Jensen

**Affiliations:** Danish Research Institute of Translational Neuroscience - DANDRITE, Aarhus University, 8000 Aarhus C, Denmark; Department of Biomedicine, Aarhus University, 8000 Aarhus C, Denmark; Danish Research Institute of Translational Neuroscience - DANDRITE, Aarhus University, 8000 Aarhus C, Denmark; Department of Biomedicine, Aarhus University, 8000 Aarhus C, Denmark; Danish Research Institute of Translational Neuroscience - DANDRITE, Aarhus University, 8000 Aarhus C, Denmark; Department of Biomedicine, Aarhus University, 8000 Aarhus C, Denmark; Danish Research Institute of Translational Neuroscience - DANDRITE, Aarhus University, 8000 Aarhus C, Denmark; Department of Biomedicine, Aarhus University, 8000 Aarhus C, Denmark; Department of Pathology, University of Washington School of Medicine, Seattle WA 98195, USA; Department of Pathology, University of Washington School of Medicine, Seattle WA 98195, USA; Department of Pathology, University of Washington School of Medicine, Seattle WA 98195, USA; Laboratory of Molecular and Chemical Biology of Neurodegeneration, School of Life Sciences Brain Mind Institute, Station 19, 1015 Lausanne, Switzerland; Centre for Misfolding Diseases,Department of Chemistry, University of Cambridge, CB2 1EW, UK; Department of Pharmacy, University of Naples, 80131, Naples, Italy; Brain Repair and Imaging in Neural Systems, Department of Experimental Medical Science, Lund University, 22184 Lund, Sweden; Laboratory of Molecular and Chemical Biology of Neurodegeneration, School of Life Sciences Brain Mind Institute, Station 19, 1015 Lausanne, Switzerland; Department of Pathology, University of Washington School of Medicine, Seattle WA 98195, USA; Department of Pathology, Zhejiang University School of Medicine and the First Affiliated Hospital, 310003 Hangzhou, China; Danish Research Institute of Translational Neuroscience - DANDRITE, Aarhus University, 8000 Aarhus C, Denmark; Department of Biomedicine, Aarhus University, 8000 Aarhus C, Denmark

## Abstract

Aggregated α-synuclein (α-syn) accumulates in the neuronal Lewy body (LB) inclusions in Parkinson's disease (PD) and LB dementia. Yet, under nonpathological conditions, monomeric α-syn is hypothesized to exist in an equilibrium between disordered cytosolic- and partially α-helical lipid-bound states: a feature presumably important in synaptic vesicle release machinery. The exact underlying role of α-syn in these processes, and the mechanisms regulating membrane-binding of α-syn remains poorly understood. Herein we demonstrate that Protein kinase R (PKR) can phosphorylate α-syn at several Ser/Thr residues located in the membrane-binding region that is essential for α-syn's vesicle-interactions. α-Syn phosphorylated by PKR or α-syn isolated from PKR overexpressing cells, exhibit decreased binding to lipid membranes. Phosphorylation of Thr64 and Thr72 appears as the major contributor to this effect, as the phosphomimetic Thr64Glu/Thr72Glu-α-syn mutant displays reduced overall attachment to brain vesicles due to a decrease in vesicle-affinity of the last two thirds of α-syn's membrane binding region. This allows enhancement of the “double-anchor” vesicle-binding mechanism that tethers two vesicles and thus promote the clustering of presynaptic vesicles in vitro. Furthermore, phosphomimetic Thr64Glu/Thr72Glu-α-syn inhibits α-syn oligomerization and completely abolishes nucleation, elongation, and seeding of α-syn fibrillation in vitro and in cells, and prevents trans-synaptic spreading of aggregated α-syn pathology in organotypic hippocampal slice cultures. Overall, our findings demonstrate that normal and abnormal functions of α-syn, like membrane-binding, synaptic vesicle clustering and aggregation can be regulated by phosphorylation, e.g., via PKR. Mechanisms that could potentially be modulated for the benefit of patients suffering from α-syn aggregate-related diseases.

Significance statement Aggregation of the nerve terminal-specific protein α-synuclein plays a pivotal role in the degenerative processes in PD, and disequilibrium between α-synucleins two physiological states, as cytosolic monomeric or vesicle-bound α-helical multimeric, respectively, appears to promote degeneration through aggregation. We report that the PKR kinase preferentially phosphorylates α-synuclein on newly identified Thr64/Thr72 residues, which lowers overall vesicle binding of α-synuclein. Yet, residually vesicle-bound phosphomimetic Thr(64,72)Glu-α-synuclein molecules increasingly adopt the vesicle-tethering “double-anchor” conformation, which promotes presynaptic vesicle clustering in vitro. Moreover, impaired oligomerization and completely abolished fibrillation and trans-synaptic inclusion pathology spreading of the phosphomimetic Thr(64,72)Glu-α-synuclein is observed. These findings challenge our interpretation of how α-synuclein is presynaptic regulated, and may open new α-synuclein-modulating strategies to alter PD progression.

## Introduction

Aggregated α-synuclein (α-syn), visualized in intracellular inclusions, is a prominent feature in the central nervous system (CNS) disorders collectively termed synucleinopathies; a group dominated by Parkinson's disease (PD), dementia with Lewy bodies (DLB) and multiple system atrophy (MSA) (1–4). α–syn is a small 140 amino acids neuronal protein that is predominantly localized in presynaptic terminals under physiological conditions. α–syn is characterized by a lysine-rich membrane-binding region (residues 1–97), which is able to fold into amphipathic α-helices and that contains an aggregation-prone hydrophobic NAC region, and an acidic C-terminus (4). Despite its natively unfolded characteristics in solution, the amphipathic region, consisting of seven imperfect KTKEGV amino acid repeats allows α-syn to bind lipid membranes, whereupon the amphipathic region coils into a single extended or broken α-helical structure, depending on the curvature of membranes (5, 6). The imperfect KTKEGV repeats are conserved across species and among the α-, β-, and γ-synuclein isoforms (7). Interestingly all the autosomal dominant mutations in the α-syn gene, which lead to familial PD, are situated close to the middle of the membrane binding domain (8–14). This suggests that lipid interactions could play a key role in pathology. In cells, α-syn exists in an equilibrium between soluble and lipid-bound states, but since α-syn does not contain a transmembrane domain or lipid anchor, the lipid-binding is mediated by the properties of the amphipathic region (15–18). The interaction with membranes is believed to play important roles in the cellular functions of α-syn, and α-syn has been proposed both to mediate clustering of synaptic vesicles through a characteristic “double-anchor” mechanism (19), and to chaperone SNAP receptor (SNARE) complexes through a direct interaction with synaptobrevin-2 at the synaptic vesicle surface (20). Moreover, membrane-, vesicle- and exosome-bound α-syn has been linked to aggregation (21–25).

Approximately 90% of aggregated α-syn found in Lewy body (LB) inclusions is phosphorylated on Ser129 (26) and recently we revealed that the ubiquitously expressed interferon-inducible serine-threonine kinase PKR, could also phosphorylate α-syn on this residue (27). Several other forms of α-syn, phosphorylated at Ser87, Tyr125, and Tyr39 have been identified in the brain and within Lewy bodies (26, 28–31). Phosphorylation on α-syn has also been established for Thr92 (30), and two other tyrosine sites, namely Tyr133 and Tyr136 (32). Furthermore, mass spectrometry (MS) analysis recently indicated that phosphorylation at Thr59, Thr64, Thr72, and Thr81 occurred in brain-derived sarkosyl-insoluble α-syn from MSA patient brain (33). Finally, Thr22, Thr44, Thr54, Thr59, Thr64, and T75 was identified upon in vitro phosphorylation of α-syn by Casein kinase 2 (CK2) (34). These findings have prompted several studies to investigate the relationship between α-syn phosphorylations and α-syn aggregation and pathology formation (27, 30, 35). Novel α-syn posttranslational modifications (PTMs) continue to emerge, and so far, nitration, dopamine modification, oxidation, ubiquitination, acetylation, O-GlcNAcylation, and glycation have all been demonstrated (36–39). Increasing evidence point to PTMs as key regulators of α-syn´s structure, functions, aggregation, subcellular localization, and clearance (35).

Here, we demonstrate for the first time that Protein kinase R (PKR) can phosphorylate α-syn on multiple Ser/Thr phosphorylation sites. We find that phosphorylation at specific residues inhibit the overall membrane-binding and aggregation of α-syn. Due to lack of antibodies the extent to which these residues are phosphorylated in biological systems has yet to be determined, but we show that phosphorylation of several newly identified Ser/Thr residues does occur in cells overexpressing α-syn. Our observations suggest intriguing new regulatory mechanisms wherein the activity of a single kinase through phosphorylation at specific residues can alter the cellular localization of α-syn, regulate vesicular clustering and influence the propensity of α-syn to undergo aggregation and trans-synaptic spreading.

## Results

### PKR kinase phosphorylates multiple residues in α-syn and disrupts α-syn lipid-binding

**Fig. 1.**
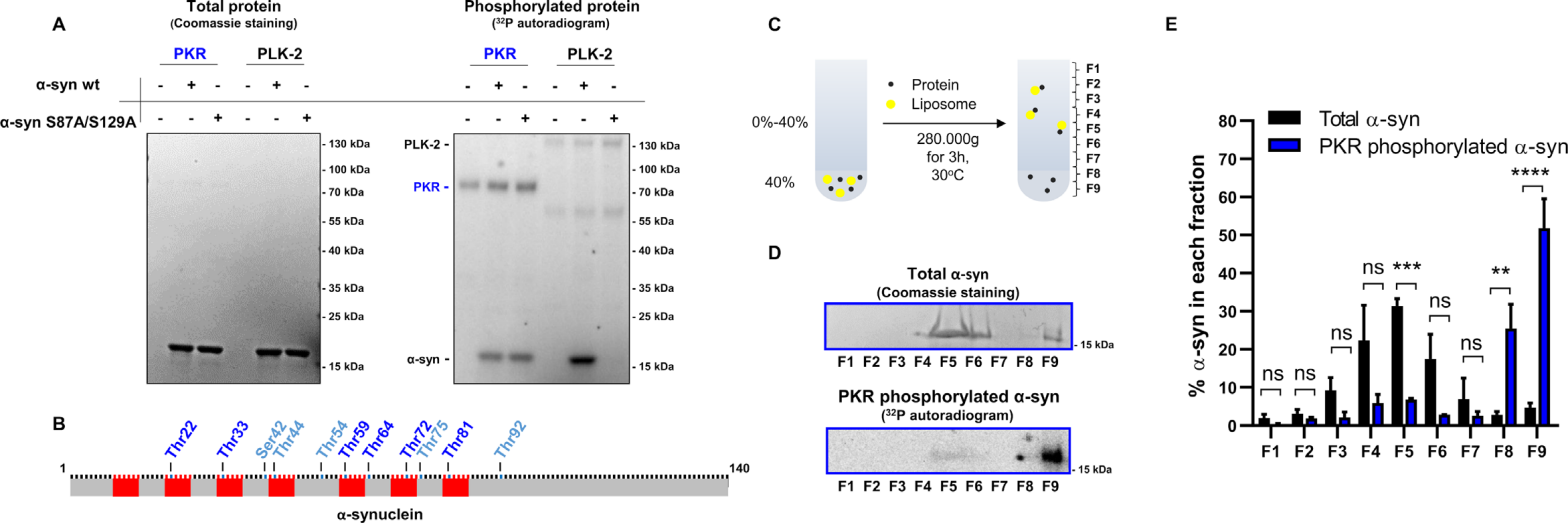
α-syn directly in vitro phosphorylated by PKR, on residues outside Ser87 and Ser129, loses the ability to bind liposomes. (A) Coomassie blue gel staining of in vitro phosphorylated samples (left panel). Recombinant human PKR (lane 1 to 3) and recombinant human PLK-2 (lane 4 to 6) was incubated for 1 h at 30°C in phosphorylation buffer (50 mM Hepes, pH 7.5 10 mM MgCl_2_, and 1 mM EGTA), with a 8:1 mixture of nonlabeled and (γ-32)-labeled Adenosine triphosphate (ATP) (10 μM final concentration). Kinases were incubated alone (lanes 1 and 4) or with substrates α-syn wt (lanes 2 and 5) or α-syn S87A/S129A (lanes 3 and 6) in a 1:50 kinase to substrate ratio. ^32^P signal from phosphorylated proteins on the Coomassie blue stained gel using standard autoradiography (right panel). Activation of kinases was evident through autophosphorylation (approximately 70 kDa for PKR (marked in blue) and 130 kDa for PLK-2 fusion protein). PKR effectively phosphorylated α-syn wt and α-syn S87A/S129A (lane 2 and 3) while PLK-2 only phosphorylated α-syn wt (lane 5). The image is representative of three independent experiments. (B) Schematic representation of the α-syn sequence with verified phosphorylations mediated by PKR (Table 1) shown in dark blue, and phosphorylations present in negative control but enhanced upon PKR incubation (Table 1) shown in light blue. The amphipathic KTKEGV regions are marked in red. (C) Schematic representation of the flotation assay. The samples in 40% sucrose are overlaid with a 0% to 40% sucrose gradient and ultracentrifuged at 280,000 x g for 3 h at 30°C. Fractions of the ultracentrifuged sample are taken and numbered from the top of the gradient. (D) Coomassie Blue gel staining of total α-syn protein on fractions from flotation assay performed with 80:20 DMPG: DMPC liposomes and α-syn S87A/S129A in vitro phosphorylated by PKR (top panel). ^32^P signal from phosphorylated α-syn S87A/S129A on the Coomassie Blue stained gel using autoradiography (bottom panel). The image is representative of three independent experiments. (E) Quantification of total α-syn levels (black bars) or PKR phosphorylated α-syn (blue bars) in each fraction of the flotation assay, based on Coomassie Blue stained gel staining and ^32^P signal, respectively. Y-axis demonstrates the percentage of the total α-syn or phosphorylated α-syn present in each fraction. Bars represent as mean ± SEM of three experiments. **P < 0.01;***P < 0.001, and ^****^P < 0.0001, based on two-way analysis of variance (ANOVA) followed by Sidak's multiple comparisons test.

Serine/threonine kinase PKR, also known as eukaryotic translation initiation factor 2-alpha kinase 2 (EIF2AK2) is considered a vital part of the cellular antiviral defense machinery and becomes increasingly active upon inflammation. Yet, active PKR has also been linked to neurodegenerative disorders such as synucleinopathies and tauopathies due to direct phosphorylation of residues associated with pathology, including the Ser129 residue of α-syn and Thr181 and Thr231 of tau (27, 40). To assess if PKR participates in the phosphorylation of additional α-syn residues besides Ser129, we performed an in vitro phosphorylation assay (Fig. 1A). Here, using radioactive ^32^P-ATP, and recombinant PKR as kinase, we assessed the phosphorylation of both α-syn wt and α-syn S87A/S129A, in which the two most well-described serine phosphorylation sites, Ser87 and Ser129, were substituted to alanine (Fig. 1A). Subsequent sodium dodecyl sulfate–polyacrylamide gel electrophoresis (SDS-PAGE) and Coomassie blue staining confirmed the purity of the proteins (Fig. 1A, left panel). Further, signal from the incorporated radioactive ^32^P revealed that PKR kinase could phosphorylate α-syn S87A/S129A to approximately the same extent as α-syn wt, which demonstrates that PKR phosphorylates other sites within α-syn than Ser87 and Ser129 (Fig. 1A, right panel). PLK-2, another Ser129 α-syn directed kinase (41), effectively phosphorylated α-syn wt, but not the α-syn S87A/S129A variant (Fig. 1A, right panel). This signifies that the two kinases exhibit different substrate specificity within α-syn where only PKR targets residues outside Ser87 and Ser129. Autophosphorylation of the kinases confirmed that both kinases were active under all assay conditions (Fig. 1A, right panel).

To identify the amino acid residues in α-syn phosphorylated by PKR, we incubated α-syn with PKR and nonlabelled ATP. α-Syn incubated with ATP without any PKR kinase served as a negative control. Subsequently, α-syn phosphopeptides were generated through trypsin and Asp-N digestion and analyzed using MS/MS (Table 1). We observed that a total of 12 residues found on 20 unique α-syn phosphopeptides were phosphorylated in the presence of PKR; namely Thr22, Thr33, Ser42, Thr44, Thr54, Thr59, Thr64, Thr72, Thr75, Thr81, Ser87, and Thr92 (Table 1). However, recombinant α-syn expressed in and purified from E. coli exhibited a nonspecific background phosphorylation on a number of these sites when incubated with ATP without PKR, including Ser42, Thr44, Thr54, Thr75, Ser87, and Thr92, but phosphorylation was increased on Ser42, Thr44, Thr54-, Ser87, and especially Thr75 residues when PKR was present. These results demonstrate that Thr22, Thr33, Thr59, Thr64, Thr72, and Thr81 are “true” PKR-dependent targets, and Ser42, Thr44, Thr54-, Thr75, and Ser87 might also be phosphorylated by PKR, at least in vitro. Although we are able to confirm PKR-mediated phosphorylation at Ser129 using Western blotting and pS129 antibodies, we did not observe Ser129 phosphorylation by MS. This is in large part because detection of this phosphorylation in the acidic C-terminal fragment remains challenging (42). Notably, of the PKR phosphorylated residues Thr22, Thr54, Thr59, Thr64, Thr72, Thr75, and Thr81 have just recently been described by other groups (33, 34) but to our knowledge, phosphorylation of Thr33 and Ser42 ha never been demonstrated.

**Table 1.** List of identified phosphopeptides derived from recombinant human α-syn wt or α-syn S87A/S129A (*n* = 3 for each variant) incubated with ATP in the presence of recombinant human PKR.

Phosphorylation sites	Phosphopeptide	α-syn wt + PKR + ATP	α-syn S87A/S129A + PKR + ATP	α-syn wt + ATP
Thr22	EGVVAAAEKpTKQGVAEAAGK	x (3/3)	x (3/3)	
Thr33	pTKEGVLYVGSK	x (2/3)	x (3/3)	
Ser42	TKEGVLYVGpSK	x (1/3)	x (1/3)	
Ser42	EGVLYVGpSKTK		x (1/3)	
Ser42	EGVLYVGpSK	x (1/3)	x (1/3)	x (1/3)
Thr44	EGVLYVGSKpTK		x (1/3)	
Thr44	EGVLYVGSKpTKEGVVHGVATVAEK		x (1/3)	
Thr44	pTKEGVVHGVATVAEK	x (2/3)	x (3/3)	x (2/3)
Thr54	EGVVHGVApTVAEK	x (2/3)	x (2/3)	x (1/3)
Thr59	EGVVHGVATVAEKpTK		x (1/3)	
Thr59	pTKEQVTNVGGAVVTGVTAVAQK		x (2/3)	
Thr64	TKEQVpTNVGGAVVTGVTAVAQK	x (2/3)	x (3/3)	
Thr64	EQVpTNVGGAVVTGVTAVAQK	x (3/3)	x (3/3)	
Thr72	TKEQVTNVGGAVVpTGVTAVAQK	x (3/3)	x (3/3)	
Thr72	EQVTNVGGAVVpTGVTAVAQK	x (1/3)	x (3/3)	
Thr75	EQVTNVGGAVVTGVpTAVAQK	x (3/3)	x (3/3)	x (1/3)
Thr75	TKEQVTNVGGAVVTGVpTAVAQK	x (3/3)	x (3/3)	
Thr81	pTVEGAGSIAAATGFVK	x (3/3)		
Ser87	TVEGAGpSIAAATGFVK	x (3/3)	N/A	x (2/3)
Thr92	TVEGAGSIAAApTGFVK	x (3/3)		x (1/3)

The phosphopeptides depicted is a mixture of nonenriched and TiO_2_ enriched samples. X symbolizes a positive hit in the analyzed sample, while numbers in parenthesis indicate the frequency of the specific phosphorylation from different MS measurements. A negative control of α-syn wt incubated with ATP in the absence of PKR was included (*n* = 3). Phosphorylation on Ser87 was not assessed for the α-syn S87A/S129A variant (N/A) due to the substitution on this site.

Interestingly, most of the identified phosphorylated residues are located in, or just adjacent to, the core amphipathic KTKEGV consensus repeat sequences essential for α-syn lipid-binding (Fig. 1B). This observation prompted us to investigate the lipid-binding of α-syn phosphorylated by PKR. Here, a flotation assay was used to separate free- and liposome-bound proteins using a density gradient as liposomes float up from the dense bottom fractions to the lighter higher fractions (Fig. 1C). Using this technique in combination with in vitro phosphorylation, we observed that the majority of the total α-syn S87A/S129A protein bound to and migrated with the liposomes into the F5 and F6 fractions of the density gradient as assessed by SDS-PAGE and Coomassie Blue staining (Fig. 1D, top panel). By contrast, the majority of the in vitro ^32^P-phosphorylated α-syn S87A/S129A remained unbound in the lowest F9 fraction of the gradient as demonstrated by autoradiography (Fig. 1D, bottom panel). This demonstrates that PKR only phosphorylates a fraction of the total amount of α-syn, but when it does, PKR-mediated phosphorylation of one or a few phosphorylation sites, apart from Ser87 and Ser129, inhibits the binding of α-syn to liposomes.

### Constitutively active PKR decreases liposome binding of cellular expressed α-syn

**Fig. 2.**
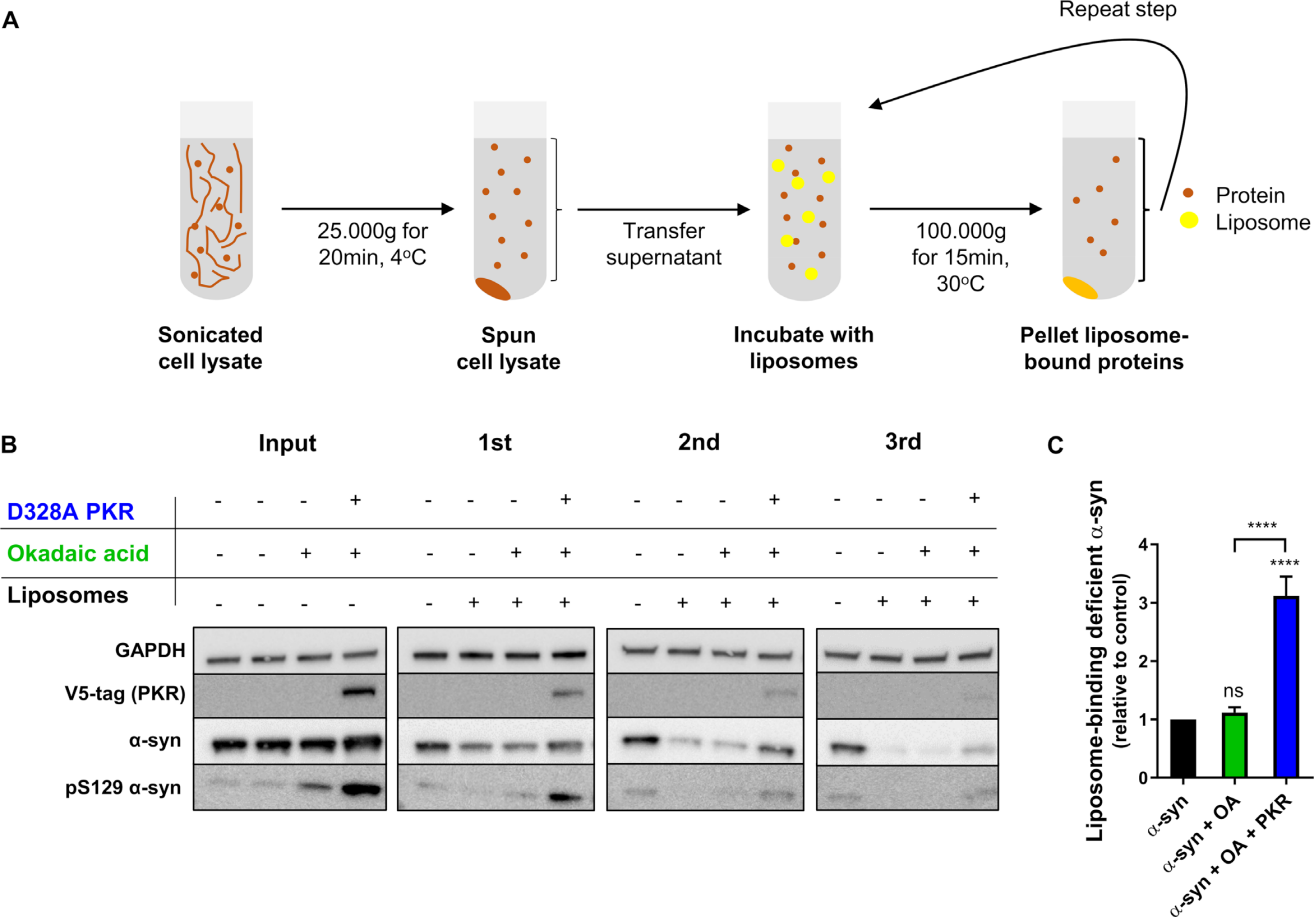
Active PKR decreases liposome binding of cellular α-syn. (A) A schematic representation of the assay to enrich the pool of proteins incapable of binding to liposomes. Cells were sonicated to create a detergent-free lysate and centrifuged 25,000 x g for 20 min at 4°C. The supernatant (input) was subsequently incubated with 80:20 DMPG: DMPC liposomes for 15 min at 37°C. To pellet liposomes and liposome-binding proteins, the samples were ultracentrifuged 100,000 x g for 15 min and nonbound proteins collected in the supernatant (1st). Two additional rounds (2nd and 3rd) of enrichment were performed on the supernatant of the 1st enrichment to produce a pure fraction of proteins deficient in liposome binding. (B) Immunoblot of supernatant from 25,000 x g centrifuged lysate (input) and 1st, 2nd, and 3rd round of enrichment of HEK 293T cells transfected with α-syn wt alone or in combination with constitutively active V5-tagged D328A PKR. Cells were treated in the absence and presence of phosph atase inhibitor, okadaic acid for the last 3 h of the incubation time, before sonication. One sample incubated without liposomes was included in the experiment as negative control. Nonliposome-binding GAPDH was used as loading control and V5 recognition used to verify PKR overexpression. Image is representative of five independent experiments. (C) Quantification of liposome-binding deficient α-syn. In all liposome-incubated samples, α-syn relative to GAPDH after 3rd enrichment was normalized to α-syn relative to GAPDH in input fraction. α-syn from untreated cells without okadaic acid treatment, and D328A PKR coexpression was set as 1. Bars represent as mean ± SEM of five experiments. ^****^P < 0.0001, based on a one-way ANOVA followed by Tukey's multiple comparisons test).

To assess if PKR also possesses the ability to decrease lipid binding of α-syn in cells, we developed a new assay designed to enrich cellular proteins incapable of binding to liposomes. Here, by incubating detergent-free cell lysates with liposomes, followed by repeated sedimentation of the liposomes and liposome-bound proteins by ultracentrifugation, we enriched a supernatant of cellular proteins deficient in binding to liposomes (Fig. 2A). To exclude the possibility that α-syn sediments in the absence of liposomes, one sample without liposomes was included. Glyceraldehyde 3-phosphate dehydrogenase (GAPDH), which does not show affinity for liposomes, was used as a loading control throughout the experiment.

Using this assay, we tested two hypotheses. First, whether a combination of overexpression of constitutively active V5-tagged D328A PKR (43) and short-term phosphatase inhibition could build up a pool of phosphorylated α-syn inside cells incapable of binding liposomes. Second, whether this pool of liposome-binding deficient α-syn could be created from endogenous kinases (short-term phosphatase inhibition alone) (Fig. 2B). We observed that inhibition of phosphatases by okadaic acid increased pSer129 α-syn levels as expected (Fig. 2B, input lane 3), but a combination of phosphatase inhibition and D328A PKR overexpression was far more effective (Fig. 2B, lane 4) (27). By comparing α-syn levels from input to 1st, 2nd, and 3rd round of enrichment, we observed that α-syn from all treatment groups gradually sedimented with the liposomes (Fig. 2B). Yet, after three rounds of enrichment, α-syn from cells coexpressing D328A PKR was three times more abundant in the supernatant (3.12 ± 0.74), compared to non-D328A PKR expressing untreated cells (Fig. 2C). This suggests that PKR kinase activity creates a pool of α-syn that fails to bind and sediment with liposomes. By contrast, short-term phosphatase inhibition did not significantly alter the liposome binding of α-syn, suggesting that the residues important for vesicle binding were not sufficiently phosphorylated by household kinases under these experimental conditions (Fig. 2C). Combined with the in vitro phosphorylation data (Fig. 1), our findings suggest a mechanism wherein activation of PKR or a kinase with similar substrate specificity and subsequent phosphorylation of specific α-syn phosphorylation sites can abrogate α-syn association to lipid-surfaces.

### Other kinases phosphorylate atypical α-syn sites, but with differential effects on liposome-binding

To determine if other kinases share PKRs ability to phosphorylate α-syn serine/threonine residues apart from Ser87 and Ser129, we used α-syn S87A/S129A as substrate and tested a library of 339 kinases (245 Ser/Thr- and 94 Tyr kinases). Here we measured the signal from incorporated ^33^P under two conditions, kinase alone (autophosphorylation), and kinase plus α-syn S87A/S129A (accumulated signal from autophosphorylation and substrate phosphorylation). If the incorporated ^33^P in the kinase plus α-syn S87A/S129A exceeded the ^33^P signal from kinase autophosphorylation, it qualified as a positive hit. Among the positive hits, we searched for kinases expressed in neuronal tissue, and found two new kinases, which target α-syn S87A/S129A, namely c-Jun N-terminal kinase 2 (JNK2) and mitogen-activated protein kinase kinase 4 (MKK4). After confirming their ability to phosphorylate α-syn S87A/S129A by auto-radiography (Supplementary Material Fig. 1A), we assessed their ability to alter the liposome-binding of α-syn S87A/S129A by phosphorylation. JNK2 appeared to disrupt liposome-binding of the phosphorylated fraction of α-syn S87A/S129A, but the weak efficiency of the kinase did not allow for proper evaluation (not shown). Contrary to the phosphorylation by PKR (Fig. 1D), α-syn S87A/S129A phosphorylated by MKK4 bound to the liposomes in the flotation assay (Supplementary Material Fig. 1B and C). This suggests that phosphorylations mediated by MKK4 does not disrupt lipid-binding of α-syn, and thereby indicates that the phosphorylation pattern mediated by PKR and MKK4 differs and cause different functional outcomes.

Based on the differential effects facilitated by PKR and MKK4 on α-syn liposome-binding, we investigated which residues within α-syn that were predominantly phosphorylated by each kinase. Using MS/MS in combination with label free quantification, we observed that the overall phosphorylation efficiency of α-syn was higher for MKK4 relative to PKR (Supplementary Material Fig. 1D), which is in agreement with ^32^P-labelled α-syn S87A/S129A (Supplementary Material Fig. 1A). Additionally, we found that the MKK4 kinase shows a strong preference for the Tyr39 site, and phosphorylation on this site is responsible for more than half (56.8%) of its total phosphorylation of α-syn (Supplementary Material Fig. 1E). This is not unanticipated since MKK4 has previously been described to also exhibit some specificity toward tyrosine residues (44). Comparing PKR and MKK-4 phosphorylation patterns on Ser/Thr residues, we observed that the Thr59, Thr64, and Thr72 α-syn residues were all phosphorylated to a higher extent by PKR (Supplementary Material Fig. 1A). Since the phosphorylation efficiency of Thr59 was low (Supplementary Material Fig. 1E and Table 1), we chose to focus on the Thr64 and Thr72 residues.

### α-syn binding to brain vesicles can be regulated by mimicking phosphorylation at Thr64 and Thr72

**Fig. 3.**
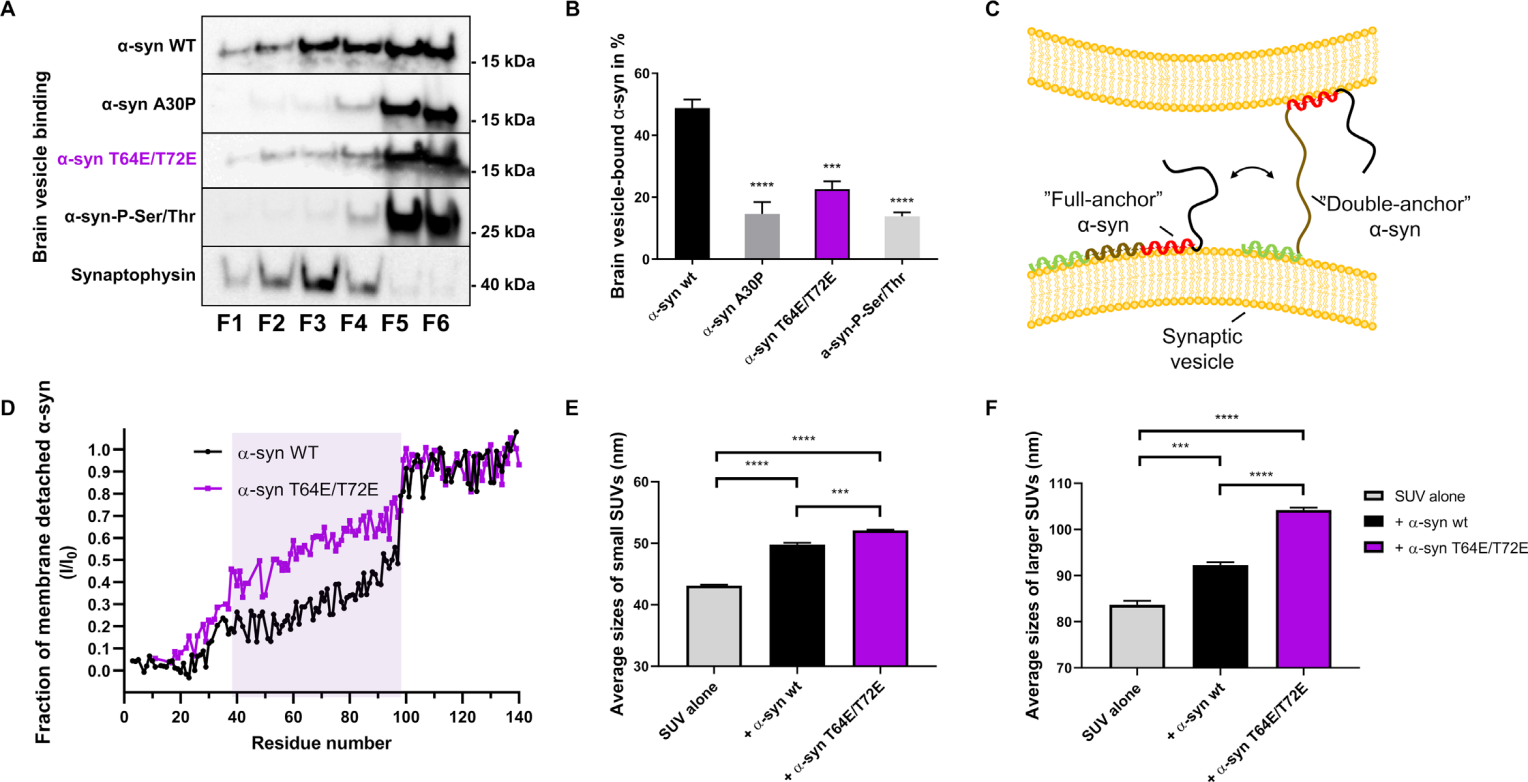
Thr64 and Thr72 in α-syn are crucial in vesicle-binding, and “double-anchor” vesicle clustering. (A) Flotation experiment using brain vesicles from α-syn KO mice. Crude mouse brain vesicles were incubated for 2 h with detergent-free lysate from HEK 293T cells transfected with α-syn wt, α-syn A30P, α-syn T64E/T72E, or α-syn-P-Ser/Thr. The solution was brought to 55% sucrose and overlaid with 35% to 55% sucrose gradient, and centrifuged for 16 h at 100,000 x g. An immunoblot of fractions from flotation assay was performed. Synaptophysin was used as a vesicle marker and positive control in the flotation assay. The α-syn-P-Ser/Thr variant migrate as a 27 kDa protein on SDS-PAGE, which is likely due to decreased SDS interaction with the introduced negatively charged residues (47). (B) Quantification of flotation experiment using mouse brain vesicles. Percent vesicle bound α-syn was determined by dividing the vesicle-bound α-syn signal (F1 to F4) to the total α-syn signal (F1 to F6) for each α-syn variant. Bars represent as mean ± SEM of three experiments. ***P < 0.001 and ^****^P < 0.0001, based on one-way ANOVA followed by Tukey's multiple comparisons test. (C) Illustrative representation of α-syn vesicle assembly through the “double-anchor” mechanism. α-syn exists in an equilibrium between two major vesicle-bound conformations, the first featuring the entire amphipathic region bound to a vesicle surface (“Full-anchor” α-syn) and the second primarily bound via the N-terminal anchor (residues 1 to 25, presented in green, “Single anchor” α-syn), which allows α-syn to seek binding to another vesicle through the central region of the protein (residues 65 to 97, presented in red) and induce vesicle clustering via the “double-anchor” mechanism (“double-anchor” α-syn conformation). (D) Nuclear magnetic resonance (NMR) chemical exchange saturation transfer (CEST) measurements providing the fraction of membrane detachment in each residue of α-syn wt (black line) and α-syn T64E/T72E (purple line) upon incubation with synaptic-like artificial vesicles. The fraction ranges from a value of 0 (minimum detachment corresponding total binding) to 1 (maximum detachment corresponding to no binding). The region associated with the strongest difference in the membrane interactions by the two α-syn constructs is highlighted in light purple (residues 38 to 97). (E) and (F) Dynamic light scattering (DLS) estimation of the size-distribution average (in nm) of the vesicles incubated alone or in the presence of α-syn wt or α-syn T64E/T72E. Grey, black, and purple bars represent the size of the initial vesicles, the vesicles incubated with α-syn wt and the vesicles incubated with α-syn T64E/T72E, respectively. Two initial sizes of vesicles were obtained by extruding the lipids through membranes with different pore diameters. The measurements show an enhanced ability of α-syn T64E/T72E to favor interaction and fusion of both small and larger synaptic- like vesicles compared to α-syn wt. Measurements were made in triplicates each consisting of 10 technical replicates, and bars represent as mean ± SEM of three three experiments. ***P < 0.001, and ^****^P < 0.0001, based on one-way ANOVA followed by Tukey's multiple comparisons test.

To test whether the negative charges of phosphate groups added to Thr64 and Thr72 might suffice in reducing α-syn binding to lipid membranes, we substituted Thr64 and Thr72 to glutamic acids, thus creating a phospho-mimetic α-syn T64E/T72E protein. Next, to investigate how these substitutions affect lipid-binding, we performed a flotation assay using brain-derived vesicles from α-syn knockout (KO) mice (Fig. 3A). Compared to artificial liposomes, surfaces of brain-derived vesicles are closer to the membranes that α-syn will encounter in a neuron. Interestingly, this assay demonstrated a weakened binding of α-syn T64E/T72E to brain vesicles compared to α-syn wt (Fig. 3A and B). The familial PD-causing α-syn A30P variant has previously been demonstrated to exhibit weakened vesicle binding properties (18), and as expected, α-syn A30P as well as α-syn-P-Ser/Thr, a construct where 11 of the newly characterized phosphorylation sites (T22/T33/S42/T44/T54/T59/T64/T72/T75/T81/T92) were substituted into phospho-mimicking residues, also failed to efficiently bind brain vesicles (Fig. 3A and B). The presence of synaptic vesicle protein synaptophysin in the less dense fractions higher in the gradient of the flotation assay confirmed that brain vesicles indeed did float up in the gradient (Fig. 3A). These data demonstrate that α-syn binding to brain-derived vesicles is lowered by Thr64 and Thr72 phosphorylation.

It has previously been demonstrated that α-syn can form a dynamic link between two synaptic-like vesicles via a “double-anchor” mechanism in which a first vesicle is tightly bound via the N-terminal region (residues 1 to 25, first anchor) and a second vesicle is bound through the central region of the protein (residues 65 to 97, second anchor) (Fig. 3C) (19). This mechanism was shown in vitro to promote the clustering of ex vivo synaptic vesicles in a calcium dependent manner (19, 45). Indeed, NMR analyses demonstrated that α-syn incubated with vesicles existed in two major vesicle-bound states, the first featuring the entire amphipathic region bound to the vesicle surface (Fig. 3C, “full-anchor” α-syn) and the second primarily bound *via* the N-terminal anchor (residues 1 to 25) and with the reminder of the protein detached from the membrane (“single-anchor” α-syn) allowing it to bind a second vesicle by the second anchor (“double-anchor” α-syn). By shifting the equilibrium from “full-anchor” toward the “single-anchor” conformation, α-syn's ability to induce vesicle clustering via the “double-anchor” mechanism (Fig. 3C, “double-anchor” α-syn conformation) can be increased. This effect was observed in the case of α-syn mutants carrying an additional negative charge in the region 65 to 97 (K80E mutation (19)) as well as when an excess cholesterol is introduced in the membrane (46).

To investigate the conformations of α-syn T64E/T72E at the membrane surface, and its role in the vesicle clustering, we used solution NMR CEST (Fig. 3D). The CEST data were processed in such a way to decouple the contribution of the local protein conformation at the membrane surface from the overall binding affinity of the protein. Using this approach, we observed that the conformations of α-syn T64E/T72E at the surface of artificial vesicles are different from those of α-syn wt, with the region 38 to 97 being less associated with the membrane surface (Fig. 3D). This likely explains the observed overall reduction in brain vesicle binding of the α-syn T64E/T72E construct (Fig. 3A and B). However, since the binding affinity of the N-terminal anchor (residues 1 to 25) appears intact, it also increases the potential for promoting vesicle clustering via the “double-anchor” mechanism. Indeed, despite the reduced overall binding affinity of the mutant toward negatively charged vesicles, the phosphomimetic substitutions at Thr64 and Thr72 induced a stronger vesicle-vesicle interaction of both small and especially larger vesicles, as probed using DLS, which is in line with the previously described K80E mutation (19) (Fig. 3E and F). These findings suggest a possible regulatory mechanism wherein threonine phosphorylations of α-syn influence its tendency to engage in the double-anchor mechanism promoting vesicle clustering in the presynapse.

### Phospho-mimicking substitution on Thr64 and Thr72 impairs de novo aggregation and elongation of α-syn seeds in vitro and in cells

**Fig. 4.**
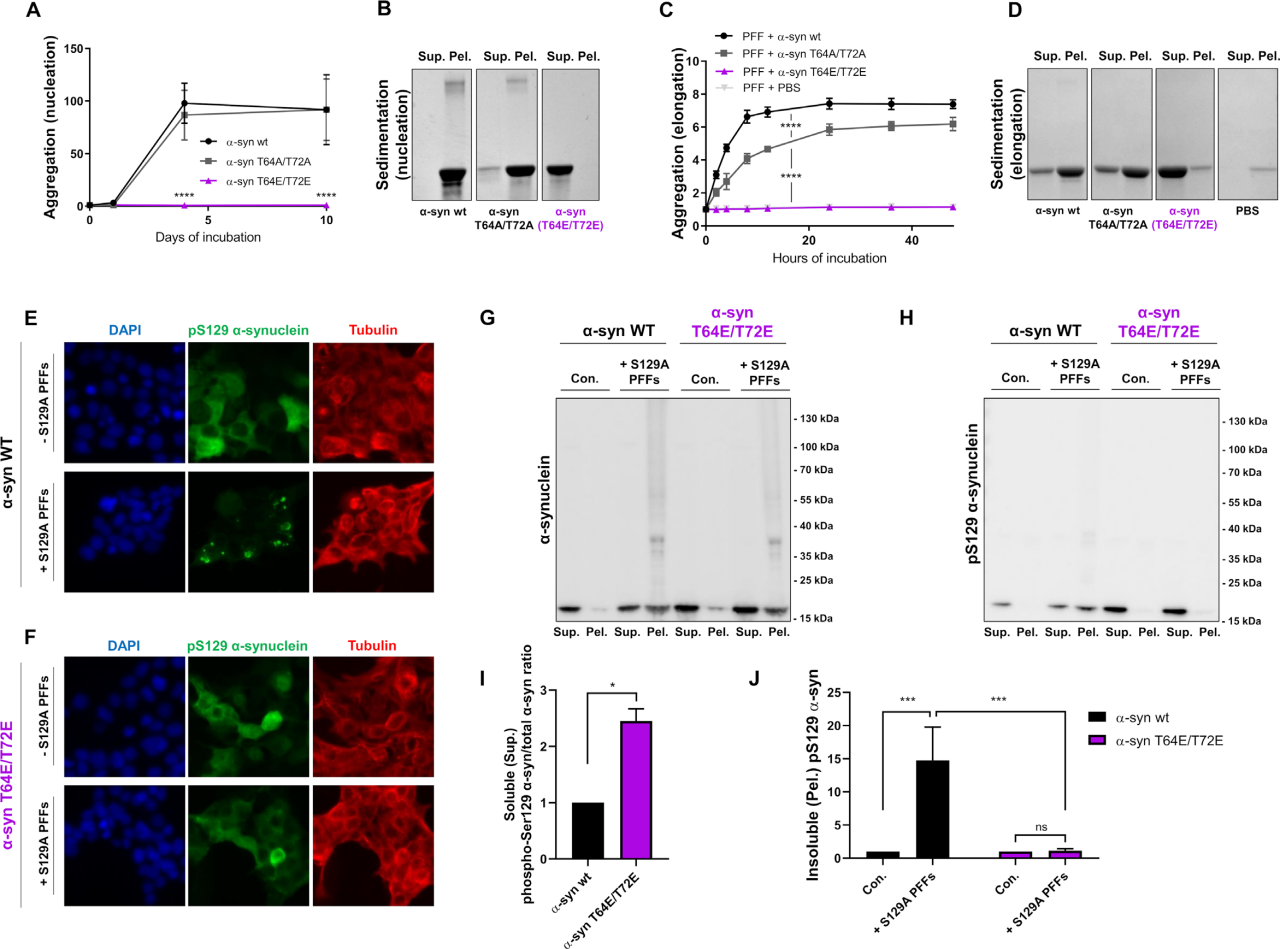
T64E/T72E phospho-mimicking α-syn shows impaired nucleation and elongation of aggregation and does not aggregate in cells. (A) Recombinant α-syn wt, α-syn T64A/T72A, and α-syn T64E/T72E were each incubated in PBS at a final concentration of 2 mg/mL at 37°C. ThT fluorescence signal was measured at day 0, 1, 4 and 10 (excitation at 450 nm and emission at 486 nm) and normalized to ThT signal from day 0 of incubation. Each sample was measured in duplicates and displayed as mean ± SD. Figure is representative of three independent experiments (*n*  =  3, ^****^P < 0.0001, based on two-way ANOVA followed by Tukey's multiple comparisons test). (B) Samples incubated for 10 days were centrifuged at 25,000 x g, 30 min at RT. Identical volumes of supernatant and resuspended pellet were subjected to SDS-PAGE and stained using Coomassie Brilliant Blue. (C) The 10% sonicated α-syn wt PFFs were incubated with 90% α-syn wt-, α-syn T64A/T72A-, α-syn T64E/T72E monomer or incubated alone (PBS) at 37°C. ThT signal was measured at 0, 2, 4, 8, 12, 24, 36, and 48 h and normalized to ThT signal from 0 h of incubation. Each sample was measured in duplicates and displayed as the mean ± SD. Figure is representative of three independent experiments (*n * =  3, ^****^P < 0.0001, based on two-way ANOVA followed by Tukey's multiple comparisons test). (D) Samples incubated for 48 h were centrifuged at 25,000 x g, 30 min at RT. Supernatant and equal volume of resuspended pellet were subjected to SDS-PAGE and stained using Coomassie Brilliant Blue. (E) HEK 293T cells were transfected with α-syn wt and 24 h post transfection exposed to 28 µg/mL sonicated S129A α-syn PFFs or left untreated. After 24 h PFF treatment, the cells were fixed and visualized using DAPI (blue), anti-phospho-S129 α-syn (green) and anti-α-tubulin (red). (F) HEK 293T cells were transfected with α-syn T64E/T72E and 24 h post transfection exposed to 28 µg/mL sonicated S129A α-syn PFFs or left untreated. After 24 h PFF treatment, the cells were fixed and visualized using DAPI (blue), anti-phospho-S129 α-syn (green) and anti-α-tubulin (red). (G) and (H) HEK 293T cells were transfected with α-syn wt or α-syn T64E/T72E for 24 h and subsequently exposed to 28 µg/mL sonicated S129A α-syn PFFs or left untreated (con.) for an additional 24 h. Cells were sonicated to create a detergent-free lysate and centrifuged 100,000 x g for 30 min at 30°C to pellet insoluble material. Supernatant and pellet fraction was analyzed in a 1:10 ratio on immunoblot using anti-α-syn (G) and anti-phospho-S129 α-syn (H) antibodies. In both (E), (F), and (H), anti-phospho-S129 α-syn was used as a marker of aggregation of cellular expressed α-syn, as the S129A α-syn PFFs do not bind this antibody. Images are representative of three independent experiments (*n* = 3). (I) Quantification of phospho-Ser129 α-syn/total α-syn ratio from supernatant fractions in cells not treated with S129A α-syn PFFs (Fig. 4G and H, lane 1 and 5). Bars represent as mean ± SEM of three experiments. *P < 0.05, based on Student's paired *t* test. (J) Quantification of insoluble pS129 α-syn (pel.) from (H) in S129A α-syn PFFs treated cells relative to untreated (con.). Bars represent as mean ± SEM of three experiments. ***P < 0.001, based on one-way ANOVA followed by Tukey's multiple comparisons test.

The relationship between α-syn membrane binding and its ability to aggregate has recently attracted attention, and its modulation has even been suggested as a potential therapeutic strategy in PD (48). However, it still remains unclear whether membrane binding can be considered beneficial or harmful in the context of aggregation (16, 49, 50). Therefore, to evaluate the impact of membrane-disrupting Thr64 and Thr72 phosphorylation on α-syn fibrillation, we compared the in vitro aggregation properties of the phospho-mimetic α-syn T64E/T72E to α-syn wt and α-syn T64A/T72A (Thr64 and Thr72 substituted to alanines) (Fig. 4A). Four days incubation of α-syn wt and α-syn T64A/T72A samples under agitating conditions induced a strongly enhanced Thioflavin T (ThT) fluorescence signal, demonstrating the presence of aggregated β-sheet-rich α-syn structures (Fig. 4A). By contrast, no increase in ThT fluorescence was observed for the α-syn T64E/T72E mutant even after ten days of incubation (Fig. 4A). Sedimentation of α-syn wt- and α-syn T64A/T72A fibrils, but not α-syn T64E/T72E, confirmed the presence of insoluble fibrillary material in the ThT positive samples (Fig. 4B). These data demonstrate that α-syn T64E/T72E is incapable of fibrillation in vitro. To examine if the phospho-mimicking substitutions on α-syn also inhibit elongation of existing fibrillary seeds we produced and purified α-syn wt preformed fibrils (PFFs) and subsequently sonication them into smaller seeds. The 10% of sonicated α-syn wt PFF seeds were incubated with 90% monomeric α-syn wt, α-syn T64A/T72A, α-syn T64E/T72E, or pure PBS. We observed that both α-syn wt and α-syn T64A/T72A actively elongated the fibrillary seeds, as evident by increased ThT signal and sedimentation (Fig. 4C and D). However, the α-syn T64E/T72E could not elongate α-syn wt PFF seeds (Fig. 4C and D).

To study how Thr64 and Thr72 phospho-mimicking substitutions might influence the oligomerization of α-syn, we used an established protocol (51) to generate soluble oligomers, which included short-term incubation of α-syn at high concentration under agitating conditions followed by centrifugation and gel filtration to separate oligomers from monomers. Here, we observed a decreased ability of α-syn T64E/T72E to form oligomers relative to α-syn wt and α-syn T64A/T72A (Supplementary Material Fig. 2A). However, similar to α-syn wt and α-syn T64A/T72A oligomers, α-syn T64E/T72E oligomers purified by gel filtration (Supplementary Material Fig. 2A) were recognized by aggregation-specific antibodies FILA and MJFR-14–6–4–2, and were capable of seeding aggregation of α-syn wt (Supplementary Material Fig. 2B and C).

To study how α-syn T64E/T72E behaved in a cellular context, we overexpressed α-syn wt or α-syn T64E/T72E in HEK 293T cells and treated the cells with α-syn PFFs substituted with an alanine at the S129-residue (S129A PFF). The S129A mutation allows us to distinguish between the externally added non-Ser129-phosphorylatable fibrils and phospho-Ser129 positive aggregates derived from cellular expressed α-syn. Here, we observed that α-syn wt and α-syn T64E/T72E both display a diffuse phospho-Ser129 specific staining in the absence of externally added PFFs (Fig. 4E and F), suggesting that both constructs are present expressed as desired. Treatment with S129A PFFs induces an expected inclusion-like phospho-Ser129 positive staining pattern in cells expressing α-syn wt (Fig. 4E). Yet, this inclusion-like staining is completely absent in cells expressing the α-syn T64E/T72E variant upon S129A PFF treatment (Fig. 4F), suggesting an impaired ability of α-syn T64E/T72E to aggregate in cells.

Using the same experimental setup, we investigated S129A PFF induced cellular α-syn aggregation by immunoblotting (Fig. 4G and H). 24 h after S129A PFF treatment, we lysed the cells by sonication and used ultracentrifugation to separate soluble from insoluble proteins. In the soluble pool we observed an increased phospho-Ser129 α-syn/total α-syn ratio of the α-syn T64E/T72E construct compared to α-syn wt (Fig. 4I). On the other hand S129A PFF treatment induced a pool of insoluble total α-syn in both α-syn wt- and α-syn T64E/T72E expressing cells (Fig. 4G, lane 4 and 8). Yet, insoluble phospho-Ser129 staining was only observed in cells expressing α-syn wt (Fig. 4H, lane 4 and 8, and Fig. 4J). This suggest that the insoluble pool of total α-syn observed in α-syn T64E/T72E expressing cells only consists of externally added non-phosphorylatable S129A PFF seeds, and that cellular expressed α-syn wt, but not the α-syn T64E/T72E variant, is incorporated into insoluble aggregates and S129-phosphorylated upon seeding.

### T64E/T72E α-syn fails to aggregate and propagate inter-neuronal α-syn pathology in organotypic hippocampal slice cultures

**Fig. 5.**
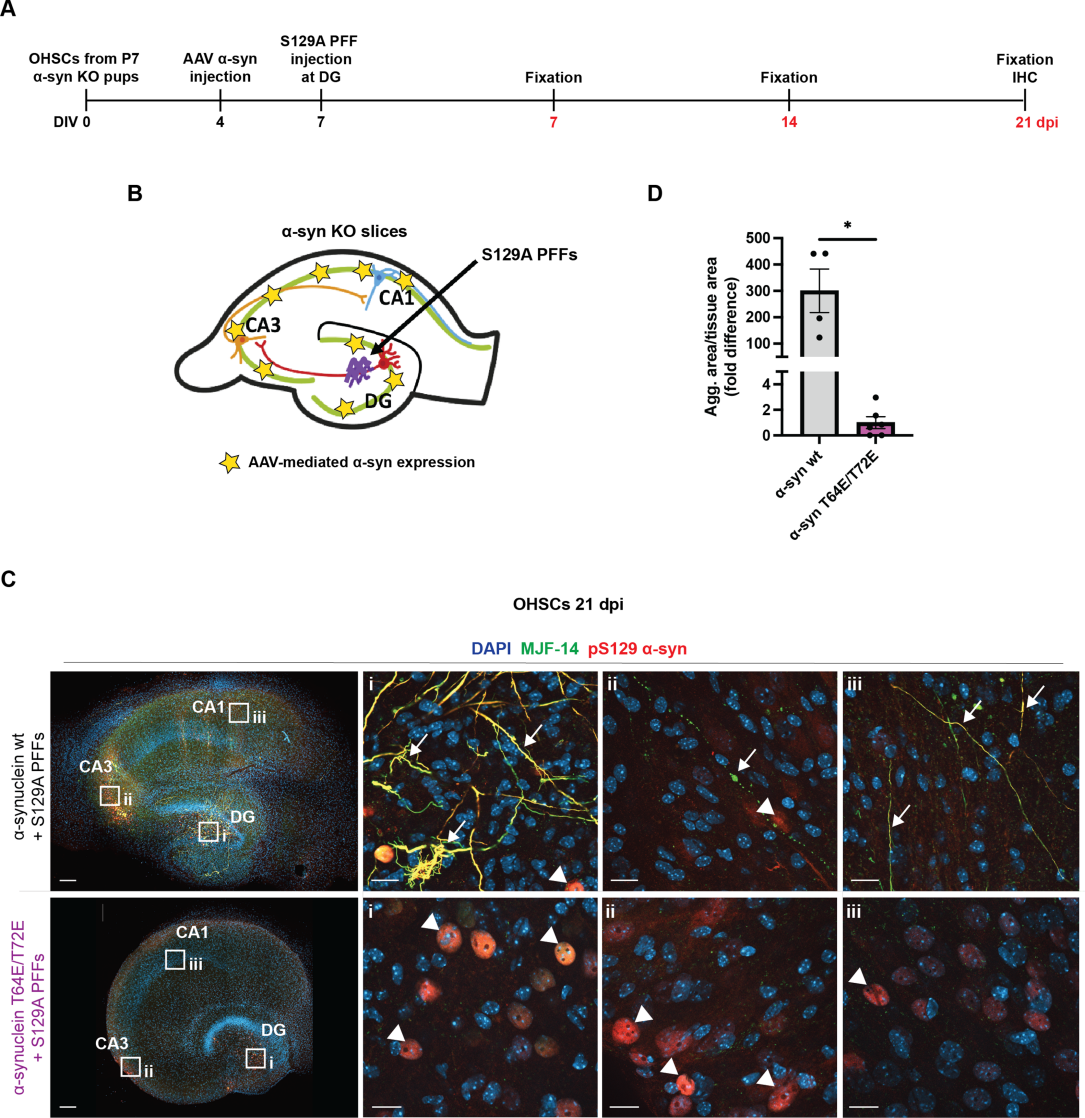
T64E/T72E phospho-mimicking α-syn fails to form inclusions and propagate pathology between neurons in OHSCs. (A) Experimental overview of OHSC setup, with AAV-mediated expression of α-syn wt or α-syn T64E/T72E at day four in vitro (DIV), S129A human α-syn PFF injection at DIV 7 and fixation of slices at 3, 7, or 21 days post injection of PFFs (dpi, marked in red). (B)A schematic overview of the hippocampal slice culture with indication of AAV injection sites (yellow stars) and PFF injection site (arrow). (C) Representative images of OHSCs expressing either α-syn wt or α-syn T64E/T72E, injected with S129A α-syn PFFs and fixed 21 dpi. Slices are stained with antibodies for aggregated α-syn (MJF-14–6–4–2, green), phospho-S129 α-syn (11A5, red), and nuclei (DAPI, blue). Arrows indicate α-syn inclusions, while arrowheads indicate nuclei with high expression of nonaggregated, S129-phosphorylated α-syn. Scale bars = 200 µM, inset = 20 µM. (D) Quantification of aggregate area normalized to tissue area (fold difference) for whole OHSCs at 21 dpi as measured by MJF-14–6–4–2 signal. Data are displayed as mean ± SEM (*n * =  4 to 6, *P  <  0.05, Student's *t* test with Welch's correction).

To expand our observations in a brain tissue model, we prepared organotypic hippocampal slice cultures (OHSCs) that allows the study of templating and interneuronal spreading of α-syn aggregate pathology between synaptically connected neurons (52, 53). In this model, human S129A α-syn PFF-injection at the dentate gyrus (DG) templates the formation of endogenous α-syn aggregates that eventually spread to the synaptically connected cornu ammonis (CA) 1 region via the CA3 region. In this particular experiment, we prepared OHSCs from α-syn KO mice where α-syn aggregates are neither formed nor propagated upon PFF-injection because no endogenous α-syn exists (52). Therefore, in order to facilitate aggregation in the DG and induce interneuronal spreading to CA3- and CA1 regions, α-syn was first expressed by injection of α-syn-encoding adeno-associated virus (AAV) in all the connected regions DG, CA3, and CA1 (Fig. 5A and B) (52). Injection of α-syn wt-encoding AAV in α-syn KO OHSCs has previously been demonstrated not to cause aggregation on its own, but only following microinjection of S129A α-syn PFFs into the DG did templated aggregation occur (52).

Using this approach, we expressed either α-syn wt or α-syn T64E/T72E in the three hippocampal regions followed by microinjection of S129A α-syn PFFs into the DG and investigated the aggregation and spreading characteristics of the two variants (Fig. 5A and B).

Microinjection of AAVs into the OHSCs gave rise to robust α-syn expression in neurons around the injection sites, with roughly equal expression levels between the two α-syn variants at 10 days post virus injection (Supplementary Material Fig. 3A). In slices expressing α-syn wt, S129A α-syn PFF injection was able to template aggregation of the AAV-expressed α-syn at the injection site in the DG, as evident through clear aggregate-specific MJF-14–6–4–2 antibody positive structures (Fig. 5C upper panel i, arrows). Further, aggregate pathology also manifested in interneuronal-connected areas CA3 to CA1 (Fig. 5C upper panel ii and iii, arrows), with substantial pathology throughout the brain slice at 21 dpi (Fig. 5C, top panel, and D). In contrast, α-syn T64E/T72E expressing slices only displayed a diffuse 300-fold lower background MJF-14–6–4–2 antibody staining with no detectable inclusions at 21 dpi of PFF treatment in all regions of the slices (Fig. 5C, bottom panel i-iii, and D). However, in these slices, phosphorylated nuclear α-syn was observed (Fig. 5C, arrowheads). This phenomenon is likely a consequence of the AAV-mediated α-syn overexpression, seeing that similar nuclear phosphorylation is observed for both α-syn variants at timepoints before aggregation is evident in the α-syn wt (Supplementary Material Fig. 3B) and using different phospho-S129 antibodies (52). Taken together, α-syn T64E/T72E is unable to form aggregate inclusions in cells and neurons and to promote interneuronal spreading of α-syn aggregate pathology.

### Cellular kinases target Thr64 and Thr72 among others α-syn residues for phosphorylation in HEK 293T cells

**Fig. 6.**
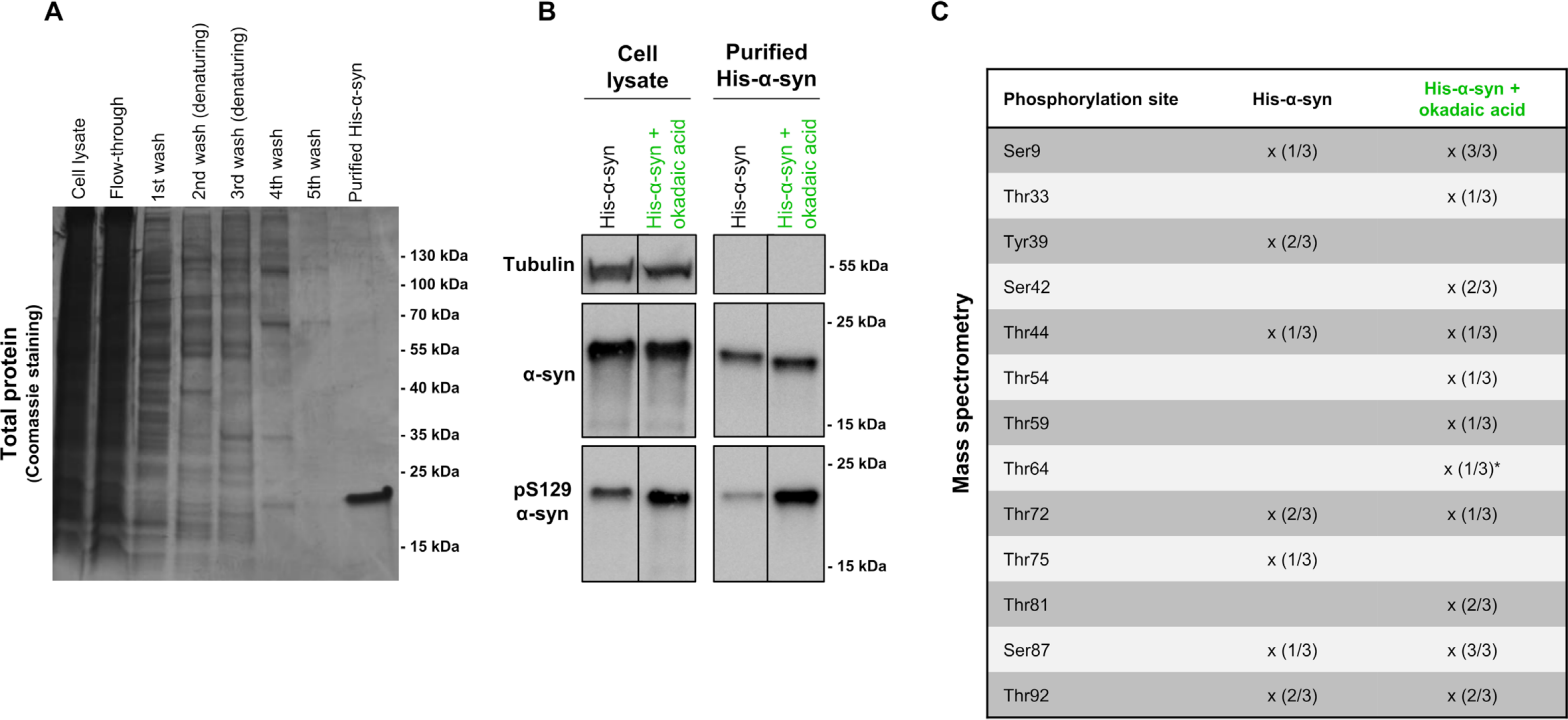
Thr64 and Thr72 among other α-syn phosphorylation sites are targeted by endogenous cellular kinases. (A) Coomassie Blue gel staining of fractions during purification of cellular His-α-syn using a His Spintrap column. During purification, all buffers were supplemented with protease- and phosphatase inhibitors to preserve phosphorylations. HEK 293T cells transfected with His-α-syn were lysed 48 h posttransfection. Cells were either treated with okadaic acid (50 nM) for 24 h prior to lysis or left untreated. Cell-lysate (2.5% of total volume), flow-through (2.5%), elute from washing steps (5% of each), and eluted purified His-α-syn (10%) fractions were loaded. (B) Cell-lysate or eluted purified His-α-syn fractions (0.5% of total volume of each) from untreated- and okadaic acid treated cells were analyzed on immunoblot using anti-β-tubulin, anti-α-syn and anti-phospho-S129 α-syn antibodies. (C) List of identified phosphorylated residues in cellular purified His-α-syn. X symbolizes a positive hit in the analyzed sample, while numbers in parenthesis indicate the frequency of the specific phosphorylation in three independent experiments. A phosphorylation was considered a hit if the confidence score exceeded 200. Asterix on Thr64 indicate a lower confidence score of 112.8. Both (A) and (B) are representative images of three independent experiments.

To investigate if endogenous cellular kinases can phosphorylate α-syn on residues identified by our in vitro phosphorylation experiments, we designed an experiment wherein α-syn was purified from HEK293T cells and analyzed by MS. To facilitate the isolation of substantial amounts of pure cellular expressed α-syn we used HEK293T cells expressing a C-terminally His-tagged version of α-syn (His-α-syn) (Fig 6A). The presence of total- and Ser129 phosphorylated His-α-syn in both crude cell lysate as well as the purified fraction was confirmed by immunoblotting (Fig. 6B). As a control, β-tubulin was found only in the crude cell lysate, suggesting that the purified fraction was indeed free from contaminating cellular proteins (Fig. 6B). To preserve phosphorylations, we exposed one subset of the cells to long-term phosphatase inhibition by okadaic acid. As expected, phosphatase inhibition elevated phosphorylation at Ser129 compared to untreated cells, which indicates that the treatment was effective (Fig. 6B). Interestingly, when assessing cellular purified His-α-syn by MS, several phosphorylations were observed. Phosphorylation at seven α-syn residues (Ser9, Tyr39, Thr44, Thr72, Thr75, Thr87, and Thr92) were evident in the untreated sample of His-α-syn, while phosphorylation at 11 α-syn residues (Ser9, Thr33, Ser42, Thr44, Thr54, Thr59, Thr64, Thr72, Thr81, Thr87, and Thr92) were detected in at least one out of three replicates of the okadaic acid treated samples (Fig. 6C), albeit Thr64 was detected with a lower confidence score. These findings demonstrate that α-syn becomes phosphorylated on both novel and previously described residues by endogenous kinases in a cellular environment, which indicate a physiological relevance of our herein described findings.

## Discussion

Ever since α-syn was discovered as a component of amyloid plaques in AD (54), its phosphorylation profile has been extensively studied both in vitro and in the brain. Nevertheless, here we successfully identify phosphorylations on α-syn serine/threonine residues that have not previously been reported. Moreover, we describe how some of these phosphorylations can change central properties of α-syn associated with both its physiological- and pathological functions.

In our opinion, several possibilities could explain how these phosphorylations have remained unnoticed. Protein phosphorylation is notoriously known for being dynamic and reversible, and often phosphorylations only last for seconds after kinase inhibition (55, 56). This suggests that proteins in cellular systems at steady state are continuously and rapidly phosphorylated and de-phosphorylated. Furthermore, as ATP-production terminates postmortem, it creates a favorable environment for phosphatases that, in contrast to kinases, work independently of ATP. Indeed, tau, a protein which also forms intracellular inclusions and shares amyloid features with α-syn, loses 50% of Thr212 phosphorylation just 40 s postmortem and this PTM is completely absent after 5 min (57). Similarly, phosphorylation at Ser129 on soluble α-syn derived from adult wt rat brains rapidly decrease after 30 min postmortem and is completely missing after 1 h (28). Patient-derived brain tissue, where postmortem delay of hours or even days is not uncommon, may therefore pose a technical caveat for detection of labile phosphorylations and will favor the detection of PTMs less accessible for de-phosphorylation, e.g., α-syn phosphorylations present within inclusions.

Cerebrospinal fluid (CSF)- and blood samples pose other sources of patient-derived α-syn where these phosphorylations could have been detected, however, α-syn is present at low concentration i.e., 1.6, 13, and 60 ng/mL in the CSF, serum and plasma, respectively, which makes discovering of low-abundant PTMs technically challenging (58).

Finally, MS on in vitro phosphorylated α-syn is often employed on α-syn phosphorylated by known Ser129-directing kinases such as members from the PLK-, GRK-, or CK kinase families. Hence, a comprehensive discovery-based analysis, using kinases with no prior α-syn-relationship, is difficult to find in published literature. In the case of the first discovered α-syn kinase, CK1, it is unsurprising that Ser129 is the preferred phosphorylation site, since Ser129 lies within its consensus recognition sequence (59). Regarding PLK-2, in line with previous findings (60), our data show that this kinase, in contrast to PKR, mediates no phosphorylation outside Ser87 and Ser129 (Fig. 1A). Similar results for GRK2 and GRK5 were previously published (61). Therefore, the research field might have overlooked phosphorylation on novel residues by utilizing kinases that preferentially target known α-syn residues and Ser129 in particular. Development of antibodies recognizing the novel phosphorylation sites identified in this manuscript could provide useful tools to study a hitherto undiscovered layer of α-syn biology.

Based on our demonstration that phosphorylation on most serine/threonine residues targeted by PKR also occur in cells without PKR overexpression (Fig. 6), we believe that PKR should be considered primarily as a proof for non Ser87, Ser129 α-syn Ser/Thr directed kinases. Many other kinases likely also play a role in the phosphorylation of residues such as Thr64 and Thr72, and considering that α-syn is physiologically enriched in nerve terminals, a better understanding of the presynaptic kinome is warranted.

When assessing the location of the Thr64 and Thr72 residues within the primary α-syn structure, both are located within the amphipathic repeats (Fig. 1B); however, based on structural models, both Thr64 and Thr72 appears to be situated adjacent to the hydrophobic membrane-binding face of the α-helix (5, 62). Nevertheless, we demonstrate a decreased binding of the α-syn T64E/T72E construct to both artificial vesicles and brain vesicles. This could indicate that not only does Thr64 and Thr72 phosphorylation bring negative charges, but they might also change the overall folding of α-syn or limit its structural flexibility: something which would also explain why α-syn T64E/T72E fails to aggregate. Besides Thr64 and Thr72, phosphorylation of Tyr39 has also previously been associated with decreased helix-2 binding to lipid vesicles, lowered membrane-binding when expressed in yeast and slower aggregation kinetics (63). Yet, MKK4, showing a strong preference for Tyr39, failed to disrupt α-syn lipid-binding, which suggests that the novel PKR-mediated phosphorylations are more potent in this regard.

Evidence demonstrates a role of α-syn in regulating presynaptic vesicle homeostasis, and α-syn has been proposed to participate both in vesicle clustering, where it helps maintain the distal reserve pool of presynaptic vesicles, but also in docking, priming, and fusion steps in exocytosis of synaptic vesicles (64). Our findings add a novel layer to the biology of α-syn membrane-interactions wherein phosphorylation on specific residues decrease the overall affinity of α-syn to vesicles. Yet, by weakening the binding of the segment containing residues 38 to 97 to synaptic vesicle-like liposomes through phosphomimetic Thr64Glu/Thr72Glu substitutions, we simultaneously increase α-syns ability form a broken-helix “double-anchor” and connect two synaptic vesicles (Fig. 3), much like the K80E α-syn mutation does (19). Docking of synaptic vesicles to the inner plasma membrane via a similar α-syn mediated “double-anchor” mechanism was recently proposed (65), and further studies could reveal if specific serine/threonine α-syn phosphorylations also alter this process. In general, it is well-described that the binding of α-syn to lipid membranes is highly dependent on the composition of phospholipids and membrane curvature (65, 66), and our data also demonstrates an increased “double-anchoring” effect of α-syn T64E/T72E on larger vesicle-like liposomes with less curvature compared to smaller ones (Fig. 3E and F). Based on our observations, one could therefore envision how phosphorylations at specific residues may shift the affinity of α-syn toward different types of cellular membranes. α-Syn has been demonstrated to bind both synaptic vesicles, ER/Golgi, and mitochondria membranes (67), and similar to the observation that familial point mutations affect α-syn localization to mitochondria-associated ER Membranes (MAM) (68), and brain vesicles (18), phosphorylations could potentially have the same effect.

Besides lipid-binding, we show that T64E/T72E α-syn mutagenesis reduces oligomer formation, and completely abolishes fibril formation, the elongation of preformed fibrils, as well as aggregation and interneuronal spreading in cells and OHSCs. Given that the NAC domain is indispensable for aggregation and Thr64/Thr72 are located herein, it is not surprising that these mutations can hamper aggregation, yet a complete loss of fibrillation is striking. When assessing high-resolution structures of wt α-syn fibrils neither Thr64 nor Thr72 appear to be present in the interface between adjacent protofibrils (69). However, according to solid-state NMR and Cryo-EM structure models, that exhibit how α-syn monomers are folded within a protofibril, Thr64, and Thr72 are located within the same area of the fibril core created by two beta-strands that span residues 59 to 72 (33, 69, 70). One model proposes that Thr64 is located close to the uncharged Val70 (70), while in the two other models the Thr64 side chain is pointing away from the other beta-strand (33, 69). More interestingly, in all three structural models, the side chain of Thr72 is located in close proximity to the side chain of Glu61 on the opposite beta-strand (69, 70). The introduction of a phosphorylation on Thr72 will therefore likely cause a repulsion between the negatively charged Glu61 and the phosphorylated Thr72 residue. Based on this, phosphorylation at Thr72 is likely more destructive for fibril formation than Thr64 phosphorylation, but more experiments are needed to draw a firm conclusion. α-syn phosphorylated at Thr59, Thr64, Thr72, and Thr81 was recently discovered in sarkosyl-insoluble material from MSA brains (33), and here it was speculated that phosphorylation at T72 may favor protofilament type PF-IIA over PF-IA. Based on our findings, it appears that Thr64 and Thr72 α-syn phosphorylation instead prevent fibrillation entirely and therefore most likely was incorporated in the MSA inclusions post aggregation or detected in nonfibrillated α-syn captured within the sarkosyl-insoluble material.

It has been suggested that α-syn exist in an equilibrium between a natively unfolded monomeric cytosolic form and a vesicle-bound α-helical multimeric species, and shifting the equilibrium toward more unfolded monomeric cytosolic α-syn appears to increase its aggregation (71). Our data suggest that abrogation of vesicle-binding do not necessarily lead to increased aggregation if this loss of binding is caused by phosphorylations at Thr64 and Thr72. Yet, although phosphorylations at Thr64 and Thr72 in itself appear harmless, they could shift the equilibrium away from aggregation-resistant vesicle-bound α-helical multimeric species and into more natively unfolded monomeric cytosolic α-syn that becomes aggregation-prone upon de-phosphorylation. We previously identified the stress activated kinase PKR as a prodegenerative kinase in a MSA cell model of soluble α-syn aggregate stress induced by overexpression of oligodendroglial protein p25α (27). In this rapid model, cytotoxicity is largely attributed to Ser129 α-syn phosphorylation as S129A α-syn substitution markedly decreases cellular degeneration (72). Therefore, the protective mechanism of PKR inhibition in our MSA model likely depends more on its role in Ser129 α-syn phosphorylation and less on its role in regulating vesicle binding. Yet, the exact relationship between PKR activity and neuropathology will have to be addressed in a completely different set of experiments. In relation to α-syn aggregate pathology, it is worth noting recent data from PD patients, which demonstrate widespread distribution of α-syn oligomers in brain areas unaffected by LB pathology, and that cognitive impairment can be associated with the load of these α-syn oligomers in the hippocampus (73).

In general we hypothesize that phosphorylation and other transient PTMs on specific α-syn residues can affect a wide array of α-syn functions, such as vesicle homeostasis and aggregation, and that such PTMs might shed light on how presynaptic vesicle biology is regulated. In support of this hypothesis multiple PTMs besides phosphorylation, including acetylation, ubiquitination, SUMOylation, and nitration, have already been demonstrated to alter the membrane binding affinity of α-syn (66).

Insertion of another PTM, O-GlcNAcylation, has previously been investigated in relation to α-syn lipid-binding and aggregation (39, 74, 75). O-GlcNAcylations represents another common intracellular PTM associated to serine and threonine residues that exhibit rapid on-off rates similar to phosphorylations (76). Nine O-GlcNAcylations have been identified within α-syn; namely Thr33, Thr44, Thr54, Thr59, Thr64, Thr72, Thr75, Thr81, and Ser87 (74), interestingly coinciding with the phosphorylation sites targeted by PKR. Although O-GlcNAcylations possess no net charge, the group is hydrophilic and adds steric hindrance once attached to proteins. Nevertheless, individual semisynthetic O-GlcNAcylations on Thr72, Thr75, Thr81, and Ser87 do not alter secondary structure of α-syn in the presence of lipid vesicles, as measured via CD spectroscopy (39, 74). Yet, precautions should be taken when comparing different techniques (CD spectroscopy versus flotation assays) using different reagents (small or large artificial liposomes versus physiological brain vesicles). The effect of O-GlcNAcylation on aggregation was more prominent. Semisynthetic O-GlcNAcylations on Thr72, Thr75, Thr81, and Ser87 all reduced nucleation and fibril elongation (39, 74). Although, the effect of Thr64 O-GlcNAcylation on α-syn aggregation remains to be investigated, these data appear to support our findings.

**Fig. 7.**
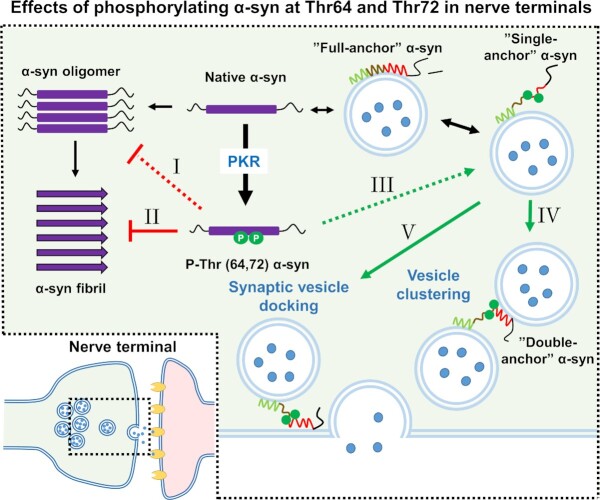
Phosphorylation of α-syn at Thr64 and Thr72 inhibits its aggregation and promotes “double-anchor” vesicle clustering. Phosphorylation of α-syn at Thr64 and Thr72 by PKR or other presynaptic kinases (I) reduces α-syn oligomerization and (II) inhibits α-syn fibrillation. Thr64 and Thr72 phosphorylation also (III) favors the “single-anchor” conformation of vesicle bound α-syn, which (IV) increases α-syn induced vesicle clustering via the “double-anchoring” mechanism, and (V) possibly increase docking of vesicles at the plasma membrane via the same mechanism. Based on this, we believe that our findings could imply a novel biology wherein presynaptic α-syn vesicle- and membrane binding and aggregation is regulated through phosphorylation.

In summary, we report how novel phosphorylation sites in α-syn can change its functional activities with respect to vesicle binding and aggregation propensity (Fig. 7). We propose a model where phosphorylation can participate in the regulation of α-syn vesicle binding from being present on one larger curvature surface to bridging smaller vesicles via the “double-anchor” mechanism through binding of N-terminal residues 1 to 25 and more distal residues 65–97, and where modifications of Thr64 and Thr72 also impair aggregation. Our cellular data, showing that several α-syn phosphorylations, including Thr64 and Thr72, is phosphorylated by endogenous kinases and their presence in brain-derived sarkosyl-insoluble α-syn in MSA patients, suggest that these phosphorylations do occur in living systems and could play a physiological role in the regulation of α-syn properties. Based on this, we believe that our findings could imply a novel biology wherein pre-synaptic α-syn vesicle- and membrane binding and aggregation is regulated through phosphorylation (Fig. 7).

## Methods

### Production and purification of recombinant α-syn

Human recombinant α-syn was extracted and purified from transformed BL21(DE3) competent cells as previously described (77). In brief, protein purification involved dialysis of proteins against 20 mM Tris pH 6.5 overnight, followed by ion-exchange chromatography on a Poros HQ50 column (Thermo Fisher Scientific) with a 0 to 2 M NaCl gradient. Next, a reverse phase-high pressure liquid chromatography purification step was performed on a Jupiter C18 column (Phenomenex, Torrance, CA, USA) in 0.1% trifluoroacetic acid with a 0% to 90% acetonitrile gradient. Isolated proteins were dialyzed in PBS pH 7.4 overnight followed by an additional dialysis step in 20 mM ammonium bicarbonate overnight. Protein concentration was determined by bicinchoninic acid (BCA) protein concentration assay (Pierce). The proteins were subsequently aliquoted, lyophilized, and stored at − 20°C until use.

### In vitro phosphorylation

Recombinant human EIF2AK2 (PKR) protein (Thermo Fisher Scientific), human PLK-2 (Thermo Fisher Scientific), MKK4 (ProQinase), or JNK2 (ProQinase) were resuspended in kinase activity buffer (100 mM Hepes, pH 7.5, 20 mM MgCl_2_, and 2 mM EGTA).

For subsequent autoradiography analysis, recombinant kinases were incubated for 1 h at 37°C with ATP (8:1 mixture of nonlabeled and (γ-32)-labeled ATP, 10 μM final concentration) in a 1:50 ratio with recombinant human α-syn wt or α-syn S87A/S129A. Each sample was subjected to SDS-PAGE, stained at room temperature (RT) using Coomassie Brilliant Blue, destained overnight (ON) at RT and left for 1 h at RT in a 35% EtOH, 3.5% glycerol solution prior to drying. The dried gel was photographed on Fuji LAS-3000 Intelligent Dark Box (Fujifilm, Japan) and developed using standard autoradiography.

For subsequent MS analysis, recombinant human α-syn wt or α-syn S87A/S129A was incubated with recombinant human EIF2AK2 (PKR) protein (Thermo Fisher Scientific) or MKK4 protein (ProQinase) in kinase activity buffer for 18 h at 37°C in a 50:1 ratio in the presence of pure, nonlabeled ATP.

### Proteomic sample preparation and phosphopeptide enrichment

Samples were prepared as previously described (78) with minor modifications. Briefly, ∼20 μg of in vitro phosphorylated α-syn sample were spiked with 2.5 μg of β-casein and 27.5 μg of bovine serum albumin, and denatured in 6 M urea in 40 mM ammonium bicarbonate (total volume: 100 μL). The proteins were reduced and alkylated by 10 mM dithiothreitol and 40 mM iodoacetamide, respectively. Samples were then diluted 6-fold in 50 mM ammonium bicarbonate with 0.5 mM of ZnSO_4_ as a phosphatase inhibitor to reduce urea concentration and digested with AspN (1:200 (w/w); Roche, South San Francisco, CA, USA) overnight at 37°C followed by trypsin (1:25 (w/w); Promega, Madison, WI, USA) overnight at 37°C.

The resulting peptides were cleaned up with MicroSpin C18 (The Nest Group, Southborough, MA, USA) chromatography prior to phosphopeptide enrichment and MS analysis. The majority (90%) of the digested sample was used for phosphopeptide enrichment using titaniumdioxide (TiO_2_) affinity purification adapted from a published protocol (79). In brief, phosphopeptides in digested samples were allowed to bind to TiO_2_ spin tips from a TiO_2_ Phosphopeptide Enrichment and Clean-up Kit (Pierce/Thermo, Rockford, IL, USA), eluted from the spin tip, and cleaned using graphite spin columns (Pierce/Thermo) according to the manufacturer's instructions. The remaining digested sample (nonenriched) was used for MS analysis directly.

### MS analysis and label-free quantification

Samples were fractionated with a nanoACQUITY UPLC (Waters, Milford, MA, USA) and analyzed with a Q Exactive Orbitrap mass spectrometer (Thermo Fisher Scientific, Waltham, MA, USA). A 30-cm-long C18 column was made in house by packing a fused silica capillary (360 μM o.d. × 75 μM i.d.) with MAGIC C18AQ 100A 5 U beads (Michrom Bioresources, Auburn, CA, USA). An electrospray ionization (ESI) tip was made by pulling the capillary prior to packing with a laser puller (Sutter Instrument Co., Novato, CA, USA). A 2-cm-long trap column was prepared similarly by packing a frit-fused silica capillary (360 μM o.d. × 75 μM i.d. × 25 cm length) with MAGIC C18AQ 200A 5 U beads. The following LC gradient was used: 0 to 60 min 5% to 35% buffer B, 60 to 61 min 35% to 80% B, 61 to 70 min 80% B, 70 to 71 min 80% to 5% B, and 71 to 90 min 5% B. Buffers A and B consisted of 0.1% (v/v) formic acid (FA) in water and 0.1% FA in acetonitrile, respectively. Mass spectra were collected with both data-dependent acquisition (DDA) and parallel reaction monitoring (PRM) modes, targeting the theoretical phosphorylation sites.

The collected data were searched against MASCOT database for identification of peptide sequences. Then the extracted ion chromatography peak (m/z mass tolerance 0.01 Da, retention time tolerance 0.5 min) was manually inspected and evaluated to ensure correct peak detection and integration. Quantification was performed using the peak areas of the identified peptides, and compared across different samples.

### Flotation experiment using liposomes

Liposomes were prepared as described by Kjaer et al. (80). In brief, DMPG (1,2-dimyristoyl-sn-glycero-3-phosphocholine) and DMPC (1,2-dimyristoyl-sn-glycero-3-phosphoethanolamine) (Avanti Polar Lipids) were mixed and dissolved in a 80:20 ratio in chloroform, and subsequently dried. The lipids were re-dissolved in PBS buffer, subjected to ten freezing-thawing cycles, and extruded through a 0.2-µM filter ten times using a mini-extruder (Avanti Polar Lipids).

For flotation assay using in vitro phosphorylated samples, recombinant human PKR (Thermo Fisher Scientific), recombinant human JNK-2 (ProQinase) or MKK-4 (ProQinase) was resuspended in kinase activity buffer and incubated for 1 h at 37°C with ATP (8:1 mixture of non-labeled and (γ-32)-labeled ATP, 10 μM final concentration) in a 1:50 ratio with recombinant human α-syn S87A/S129A. Flotation was performed in a 10:1 liposome: protein ratio. The mixture was incubated for 1 h at 37°C with agitation, brought to 40% sucrose, and placed into a 0% to 40% sucrose gradient. The samples were ultracentrifuged at 280,000 x g for 3 h at 30°C (SW 50.1 Beckman rotor) to separate liposome-bound proteins. Following ultracentrifugation, the gradient was divided into fractions, collected from the top, precipitated with 30% trichloroacetic acid (final concentration), washed in ice-cold EtOH, dissolved in loading buffer and subjected to SDS-PAGE, stained at RT with using Coomassie Brilliant Blue, destained ON at RT and left for 1 h at RT in a 35% EtOH, 3.5% glycerol solution prior to drying. The dried gel was photographed on a Fuji LAS-3000 Intelligent Dark Box (Fujifilm, Japan) and developed using standard autoradiography.

### Cell culture and treatment

HEK 293T cells were maintained at 37°C under 5% CO_2_ and grown in DMEM (Lonza) supplemented with 10% fetal calf serum, 50 U/mL penicillin, and 50 µg/mL streptomycin. For experiments, cells were seeded 24 h before transfection. To produce detergent-free cell lysates, cells were lysed by sonication (4 × 10 strokes using a Branson Ultrasonics Sonifier S-250A, duty cycle 80 and output control 6) in a water bath continuously renewed by cold running water to prevent heating. The samples were centrifuged at 25,000 x g for 20 min at 4°C and the supernatant was collected as detergent-free lysate. To inhibit phosphatases, cells were treated okadaic acid for 3 h in a final concentration of 100 nM or for 24 h in a final concentration of 50 nM.

### Plasmids and transfection

For transfection, pcDNA3.1 zeo(-) plasmids encoding human α-syn wt, α-syn A30P or human V5-tagged PKR construct pcDNA3-PKR D328A (43), as well as pHR'CMV plasmids encoding α-syn-P-Ser/Thr (T22D/T33D/S42D/T44D/T54D/T59D/T64D/T72D/T75D/T81D/T92D) and α-syn T64E/T72E was used. The pcDNA3-PKR D328A construct was a kind gift from Dr. Ganes C. Sen (Cleveland Clinic, Lerner Research Institute). All constructs were confirmed by sequencing. Transient transfections were performed with Lipofectamine 3000 (Thermo Fisher Scientific) transfection reagent according to the manufacturer's protocol.

### Sedimentation assay to enrich for liposome-binding deficient proteins

For enrichment of liposome-binding deficient proteins, HEK 293T cells were resuspended in PBS supplemented with protease- and phosphatase inhibitors (25 mM β-glycerolphosphate, 1 mM Na_3_VO_4_ and 1 mM NaF) and lysed by sonication (4 × 10 strokes using a Branson Ultrasonics Sonifier S-250A, duty cycle 80 and output control 6) in a water bath continuously renewed by cold running water to prevent heating. The lysates were centrifuged at 25,000 x g for 20 min at 4°C and the supernatant was collected for further enrichment. The protein concentration was adjusted to 2 mg/mL and the lysates incubated with 2 mg/mL 80:20 DMPG: DMPC liposomes for 15 min at 37°C with agitation. To pellet liposomes and liposome-bound proteins the samples were centrifuged 100,000 x g for 15 min. Liposome-binding deficient proteins were collected in the supernatant (1st enrichment). Two additional rounds of enrichment using the same liposome concentration was performed on the supernatant of the 1st enrichment to produce a pure fraction of liposome-binding deficient proteins. Each sample was subjected to SDS-PAGE and immunoblotting using blocking buffers supplemented with phosphatase inhibitors to prevent de-phosphorylation. Densitometry was carried out in ImageJ software (NIH, USA); all protein bands were quantified relative to GAPDH.

### Immunoblotting

For immunoblotting, samples were dissolved in loading buffer (4% SDS, 40% glycerol, 1% bromophenol blue, and 50 mM Tris, pH 6.8 supplemented with 0.5 M dithioerythritol), denatured at 95°C for 5 min before being resolved on 10% to 16% polyacrylamide gels and transferred onto polyvinylidine difluoride (PVDF) membranes (GE Healthcare, UK). To enhance visualization of α-syn, the membranes were fixed in 4% paraformaldehyde (PFA) in PBS for 30 min followed by boiling in PBS for 5 min as previously described (81). PVDF membranes were blocked in 5% nonfat milk dissolved in TBST (10 mM Tris, pH 7.4, 150 mM NaCl, 0.1% Tween-20) for 1 h at RT, incubated with primary antibodies overnight at 4°C, washed, and incubated with horseradish peroxidase (HRP) conjugated secondary antibodies (DAKO, Denmark) for 1 h at RT. Proteins were visualized with enhanced chemiluminescence using a Fuji LAS-3000 Intelligent Dark Box (Fujifilm, Japan).

### Kinase screen

A radiometric protein kinase assay (KinaseFinder, ProQinase) was used to discover additional Ser/Thr kinases that could phosphorylate α-syn S87A/S129A. In short, α-syn S87A/S129A was dissolved in ProQinase activity buffer (60 mM HEPES, pH 7.5, 3 mM MgCl_2_, 3 mM MnCl_2_, 3 μM sodium orthovanadate, 1.2 mM DTT, 1 μM [^33^P]-ATP) together with 1–300 ng protein kinase. All PKC assays (except the PKC-mu and the PKC-nu assay) contained 1 mM CaCl_2_, 4 mM EDTA, 5 μg/mL phosphatidylserine, and 1 μg/mL 1,2-dioleyl-glycerol. The CAMK1D, CAMK2A, CAMK2B, CAMK2D, CAMK4, CAMKK1, CAMKK2, DAPK2, EEF2K, MYLK, MYLK2, and MYLK3 assays contained 1 μg/mL calmodulin and 0.5 mM CaCl_2_. The PRKG1 and PRKG2 assays contained 1 μM cGMP. The DNA-PK assay contained 2.5 μg/mL DNA. The reaction cocktails were incubated at 30°C for 60 min. The incorporated ^33^P was detected by the addition of a scintillator and determined with a microplate scintillation counter.

### Flotation experiment using mouse brain vesicles

Mouse brain vesicles were prepared as described in Jensen et al. (18) with small modifications. In brief, one cerebral hemisphere from an adult α-syn KO mice was dounce-homogenized in 1:5 (w/v) ratio of ice-cold homogenization buffer [5 mM dithiothreitol, 2 mM EDTA, 9% sucrose, 25 mM MES, pH 7.0, in the presence of complete protease inhibitor mix (Roche)]. Nuclei and debris were removed by centrifugation 5 min, 1000 x g at 4°C. The crude vesicle fraction was isolated by ultracentrifugation of the supernatant at 100,000 x g for 1 h at 4°C. The resulting pellet was resuspended by dounce homogenization in a homogenization buffer. Vesicle binding was performed by 2 h incubation of 50 μL of resuspended vesicles (approximately 15 µg/µL) with 50 µL detergent-free lysate from HEK 293T cells (approximately 4 µg/µL) transfected with different α-syn constructs. The solution was brought to 55% sucrose in a volume of 0.5 mL, placed into a 2.5-mL ultracentrifuge tube, and overlaid with 1.5 mL of a 55% to 35% sucrose gradient. Flotation was carried out for 16 h at 100,000 x g in a TLS-55 swinging rotor. Following ultracentrifugation, the gradient was divided into fractions, collected from the top, precipitated with 30% trichloroacetic acid (final concentration), and immunoblotted as described above. Percent vesicle bound α-syn was determined by dividing the vesicle-bound α-syn signal (F1 to F4) to the total α-syn signal (F1 to F6).

### CEST

We employed CEST NMR to probe the equilibrium between membrane unbound and membrane bound states of αS at a residue-specific resolution. CEST were based on ^1^H-^15^ N HSQC solution spectra by using constant wave saturation (either 350 or 170 Hz) in the ^15^ N channel, applied off-resonance up to 28 kHz (−28, −21, −14, −9, −5, −3, −1.5, 0, 1.5, 3, 5, 9, 14, 21, and 28 kHz). The method exploits the saturation of broad spectroscopic transitions in the membrane-bound state (undetectable) while leaving the resonances of the cytosolic state (detectable) virtually unperturbed (i.e., except when the offset is set to 0 kHz). The saturation induced in the membrane-bound state is subsequently detected in the form of signal attenuation of the free state as a result of chemical exchange. NMR experiments were carried out at 10°C on a Bruker spectrometer operating at ^1^H frequencies of 700 MHz equipped with triple resonance HCN cryo-probe. An additional spectrum, saturated at −100 kHz, was recorded as a reference. The spectra measured at each offset were analyzed by normalizing the signal attenuation in such a way that the strongest and weakest saturations observed adopted values of 0 and 1, respectively. This normalzsed index, which was averaged across the offsets ranging from −5.0 to +5 kHz (excluding 0 kHz), was denoted as the fraction of membrane detachment.

### DLS

DLS measurements of vesicle size distributions were performed using a Zetasizer Nano ZSP instrument (Malvern Instruments, Malvern, UK) with backscatter detection at a scattering angle of 173°. The viscosity (0.8882 cP) and the refractive index (1.330) of water were used as parameters for the buffer solution, and the material properties of the analyte were set to those of the lipids (absorption coefficient of 0.001 and refractive index of 1.440). SUVs were used at a concentration of 0.05% in these measurements and the experiments were performed at 25 °C. The acquisition time for the collection of each dataset was 10 s and accumulation of the correlation curves was obtained using ten repetitions. Each measurement was repeated ten times to estimate the SD and average values of the centres of the size distributions.

### Aggregation- and sedimentation assay (nucleation)

Lyophilized recombinant α-syn wt, α-syn T64A/T72A, and α-syn T64E/T72E was re-suspended in sterile PBS (Gibco) to a final concentration of 2 mg/mL and placed in an Eppendorf Thermotop shaker at 37 °C, 1050 rpm. To test for aggregation, a small fraction from each sample was withdrawn at day 0, 1, 4 and 10 and tested for the Thioflavin-T (ThT) signal. In brief, 100 µL of 40 µM ThT dissolved in measure buffer (90 mM glycine, pH 8.6) was added to 10 µL of each protein sample and incubated for 5 min at RT before measuring ThT signal on an EnSpire 2300 Multilabel Plate Reader (PerkinElmer Life Sciences) (excitation at 450 nm and emission at 486 nm). The signal was normalized to the ThT signal from 0 days of incubation.

For sedimentation, samples incubated for 10 days were centrifuged at 25,000 x g, 30 min at RT, and the supernatant was transferred to a new tube. The pellet was resuspended in an equal volume of PBS. Same volume of each sample was subjected to SDS-PAGE, stained at RT using Coomassie Brilliant Blue, destained ON at RT and left for 1 h at RT in a 35% EtOH, 3.5% glycerol solution prior to drying. The dried gel was photographed on Fuji LAS-3000 Intelligent Dark Box (Fujifilm, Japan).

### Aggregation- and sedimentation assay (elongation)

PFFs were prepared as described above from monomeric α-syn wt. PFFs were separated from nonfibrillary residual protein by 25,000 x g centrifugation and washed twice before resuspension in PBS. PFFs were sonicated (20 min, using a Branson Ultrasonics Sonifier S-250A, duty cycle 30 and output control 3, in a water bath continuously renewed by cold running water to prevent heating) to produce a uniform population of elongation-prone seeds and the concentration adjusted to 2 mg/mL (82). Lyophilized recombinant monomeric proteins were resuspended in sterile PBS (Gibco) and incubated with sonicated PFFs in a 90:10 ratio and placed in an Eppendorf Thermotop shaker at 37°C, 1050 rpm. To test for aggregation, a small fraction from each sample was withdrawn at 0, 2, 4, 8, 12, 24, 36,  and 48 h and measured as described above. The signal was normalized to ThT signal from 0 h of incubation. Sedimentation was performed on samples incubated for 48 h as described above.

### Oligomer preparation

α-syn oligomers were prepared as previously described (51). Briefly, 12 mg/mL lyophilized recombinant α-syn monomer was dissolved in sterile PBS (Gibco) and incubated at 37°C at 900 rpm for 5 h in an Eppendorf Thermotop shaker. After incubation, the sample was centrifuged at 12000 x g for 5 min and the supernatant was loaded onto a Superdex 200 10/300 GL column (GE Healthcare). The column was eluted with PBS at a flow rate of 0.5 mL/min. Oligomers were collected between 18 to 22 min and monomers after 38 to 43 min.

### Dot-blot

Dot blotting was performed as previously described (83) where 100 ng of protein was blotted on a nitrocellulose membrane (Hydrobond-C Extra, GE Healthcare). All membranes were blocked in nonfat milk (TBS with 5% nonfat milk, 0.05% Tween 20 and 0.02% sodium azide) for 30 min at RT followed by incubation with primary antibodies overnight. Membranes were washed in TBS-T (TBS with 0.1% Tween) and incubated with secondary antibodies for 1.5 h. Membranes were washed, developed using ECL reagent (Pierce, Thermo Scientific), and subsequently imaged on a LAS-3000 imaging system (Fuji).

### Seeding of aggregation using oligomers

α-syn oligomers were prepared and purified as described above from either recombinant monomeric α-syn wt, α-syn T64A/T72A or T64E/T72E. Recombinant monomeric α-syn wt was then incubated at 37°C in sterile PBS (Gibco) in a concentration of 2 mg/mL alone or in the presence of 0.2% α-syn wt, α-syn T64A/T72A or T64E/T72E oligomers. To test for aggregation, a small fraction from each sample was withdrawn at 0, 18, 24, 42, 66, 72, 90, and 100 h and measured as described above. The signal was normalized to ThT signal from 0 h of incubation.

### Seeding of cellular aggregation

For immunocytochemistry, HEK 293T cells were seeded onto poly-L-lysine-coated coverslips and transfected with either α-syn wt or α-syn T64E/T72E for 24 h and subsequently exposed to 28 µg/mL S129A α-syn PFFs (sonicated as above) or left untreated. The S129A PFFs are made from monomeric substituted S129A α-syn and cannot be phosphorylated on the S129 residue. After 24 h of PFF treatment, the cells were washed in PBS and fixed in 4% PFA for 10 min, washed in PBS, and incubated briefly in 15 mg/mL NH_4_Cl in PBS. Coverslips were blocked and incubated with primary and secondary antibodies in the blocking buffer [PBS, 3% bovine serum albumin (BSA), 0.1% saponin], with washings in between in PBS with 0.1% saponin and finally double-distilled H_2_O, before mounting with Fluorescent Mounting Medium (DAKO, S3023) on glass slides and left in dark ON at 4°C.

For immunoblotting HEK 293T cells were transfected with α-syn wt or α-syn T64E/T72E for 24 h and subsequently exposed to 28 µg/mL sonicated S129A α-syn PFFs or left untreated for an additional 24 h. Cells were washed and harvested in PBS with phosphatase inhibitors and sonicated to create a detergent-free lysate. The cell lysate was centrifuged 100,000 x g for 30 min at 30°C to pellet insoluble material. The supernatant and pellet fraction was analyzed in a 1:10 ratio.

### Adeno-associated virus-mediated α-syn expression and seeding in organotypic slices from α-syn KO mice

Organotypic hippocampal slice cultures (OHSCs) were created from P7 α-syn KO mice according to Stoppini et al. (84), with slicing and culture media compositions as previously reported (52). At day 4 in vitro (DIV), adeno-associated virus (AAV) with either α-syn wt or α-syn T64E/T72E was microinjected into the slices to facilitate α-syn expression before microinjection of S129A α-syn PFFs at DIV 7, as described in (52). In brief, a microinjection setup with a microscope in a LAF bench, a PulsePal v2 (#1102) to control injection and injection needles pulled on a Sutter P-1000 was employed. Production and purification of AAV-vectors were done as previously described (52, 85), with final titers of approx. 3 × 10^13^ genome copies/mL. For AAV-mediated α-syn expression, one shot of virus was given at three sites in each of the regions dentate gyrus (DG), cornu ammonis (CA) 1 and 3 under microscopic guidance (Fig. 6B). To induce aggregation, 2 × 6 shots of sonicated human S129A α-syn PFFs were injected into the DG, after which media was replaced with fresh, preheated culture media to remove any PFFs deposited in the media. To verify proper AAV-mediated α-syn expression, some slices were only injected with AAVs and fixed 10, days post virus injection. OHSCs injected with both AAV and PFFs were fixed at 3, 7, or 21 days post injection (dpi) (Fig. 6A).

Fixation was done in 4% PFA for 25 min, washed in PBS, followed by 5 min in 20% methanol in PBS and another PBS wash. For staining, OHSCs were permeabilized for 3 h in 0.5% triton X-100 in PBS, followed by 3 h of blocking in 10% BSA and primary antibody incubation in 5% BSA/PBS ON at 4°C. Unbound primary antibody was removed by 3x washing in 1x TBS + 0.3% triton X-100 and followed by secondary antibody incubation for 3 h at RT in 5% BSA/PBS, protected from light. After final washes, slices were mounted using fluorescent mounting medium (DAKO, S3023) and left to dry in the dark ON at 4°C. Primary antibodies used were rabbit antihuman α-syn (MJFR1, Abcam, ab138501, 1:5000), rabbit aggregation-specific anti-α-syn (MJF-14–6–4–2, Abcam, ab209538, 1:25,000) and mouse anti-phospho-Ser129-α-syn (11A5, kindly provided by Imago Pharmaceuticals, 1:10,000). Secondary antibodies were AlexaFluor-conjugated anti-rabbit 488 (Invitrogen, A11008) and anti-mouse 568 (Invitrogen, A11004), diluted 1:2000 and supplemented with DAPI to stain nuclei (TH.GEYER, 5 µg/mL).

### Purification of cellular expressed His-tagged α-syn for MS analysis

HEK 293T cells were transfected with C-terminally His-tagged α-syn (Sino Biological Inc.) for 48 h prior to lysis in RIPA lysis buffer [50 mM Tris, pH 7.4, 150 mM NaCl, 1% Triton X‐100, 0.5% sodium deoxycholate, 0.1% sodium dodecyl sulfate, 1 mM EDTA, 1 × cOmplete protease inhibitor cocktail (Roche)] with phosphatase inhibitors (25 mM β‐glycerolphosphate, 1 mM Na_3_VO_4_, 10 mM Na‐pyrophosphate, 1 mM NaF) on ice for 30 min and lysates were centrifuged at 25,000 x g for 30 min at 4°C. The resulting supernatant was saved as the cell-lysate. Cell-lysate was loaded on a His Spintrap column (Cytiva, Sigma–Aldrich) and purified according to manufacturer, but with modifications to increase the purity of eluted His-α-syn. We included several denaturing washing steps in the following order: 1st wash (20 mM sodium phosphate, 500 mM NaCl, and 10 mM imidazole, pH 7.4), 2nd wash (20 mM Tris-HCl, 8 M urea, 500 mM NaCl, 5 mM imidazole, pH 8.0 + 5 mM DTE), 3rd wash (20 mM Tris-HCl, 8 M urea, 500 mM NaCl, 5 mM imidazole, pH 8.0 + 1% SDS), 4th wash (20 mM sodium phosphate, 500 mM NaCl and 40 mM imidazole, pH 7.4) and 5th wash (20 mM sodium phosphate, 500 mM NaCl and 80 mM imidazole, pH 7.4). His-α-syn was eluted in 20 mM sodium phosphate, 500 mM NaCl, and 500 mM imidazole, pH 7.4. All buffers were supplemented with protease- and phosphatase inhibitors (25 mM β-glycerolphosphate, 1 mM Na_3_VO_4_ and 1 mM NaF).

### MS analysis of cellular expressed His-α-syn

Proteins were extracted and digested in-solution, based on a dual-digestion protocol, i.e., either with trypsin (100 ng/µL in 10 mM HCl) or GluC (100 ng/µL in 10 mM HCl). Briefly, 5 µL of each sample were mixed with either 40 µL of 10 mM Tris/2 mM CaCl2, pH 8.2, and 5 µL trypsin, or 50 mM ammonium bicarbonate buffer pH 8 and 5 µL GluC. In both enzymatic approaches, digestion was performed at 37°C overnight. As a next step, peptides were dried in a SpeedVac and dissolved 20 µL ddH2O + 0.1% formic acid. Before the injection, the sample digests were diluted 1:1, and 1µL were injected onto a nanoAcquity UPLC coupled to a Q-Exactive mass spectrometer (Thermo Fisher Scientific) as follows.

MS analysis was performed on a QExactive mass spectrometer (Thermo Scientific) equipped with a Digital PicoView source (New Objective) and coupled to an M-Class UPLC (Waters). Solvent composition at the two channels was 0.1% formic acid for channel A and 0.1% formic acid, 99.9% acetonitrile for channel B and the column temperature was 50°C. For each sample, 1 μL of peptides were loaded on a commercial ACQUITY UPLC M-Class Symmetry C18 Trap Column (100 Å, 5 µM, 180 µM x 20 mm, Waters) followed by ACQUITY UPLC M-Class HSS T3 Column (100 Å, 1.8 µM, 75 µM X 250 mm, Waters) and elution was performed using a flow rate of 300 nL/min. A gradient from 5% to 35% B in 90 min and 35% to 45% B for an additional 10 min was applied. Next, the column was cleaned after the run by increasing to 95% B and holding 95% B for 10 min before re-establishing the loading condition.

The mass spectrometer was operated in a data-dependent mode (DDA), with an inclusion list of α-syn + 2 and + 3 precursor ions. Full-scan MS spectra (300 to 2000 m/z) were acquired at a resolution of 70,000 at 200 m/z after accumulation to a target value of 6,000,000. Data-dependent MS/MS were recorded followed by HCD (higher-energy collision dissociation) fragmentation on the most intense signals per cycle. HCD spectra were acquired at a resolution of 35’000 using a normalized collision energy of 25 and a 12 maximum injection time of 110 ms. The automatic gain control (AGC) was set to 50,000 ions. Charge state screening was enabled. Singly, unassigned, and charge states higher than seven were rejected. Only precursors with an intensity above 9,100 were selected for MS/MS. Precursor masses previously selected for MS/MS measurement were excluded from further selection for 30 s, and the exclusion window was set at 10 ppm. The samples were acquired using internal lock mass calibration on m/z 371,010 and 445.,200. The MS proteomics data were handled using the local laboratory information management system (LIMS) (86).

The acquired mass spectra data were analyzed via Byonic 3.4 software (Byonic, Protein Metrics, USA) and peptide lists searched against the human UniProt Fasta database with the Mascot search engine. All searches were performed using trypsin or GluC digestion, and Oxidation (M), Acetylation (N-term), phosphorylation (serine/threonine/tyrosine), acetylation (N-term, K), methylation (R), and ubiquitination GlyGly (K) were defined as variable modifications.

### Antibodies

Anti-glyceraldehyde-3-phosphate dehydrogenase (GAPDH, MAB374 Merck Millipore), anti-α-tubulin (T9026 Sigma–Aldrich Co.), anti-α-syn (ASY-1, affinity-purified rabbit antibodies toward human α-syn), phospho-Ser129-α-syn (11A5), rabbit-anti-α-syn (phospho S129) (Abcam, catalog no. ab51253) anti-V5 tag antibody (SV5-Pk1, Abcam) and anti-Synaptophysin (YE269, Abcam) were used in accordance with the manufacturers' recommendations. HRP-conjugated rabbit immunoglobulins (#P0217, Dako) and secondary HRP conjugated mouse immunoglobulins (#P0260, Dako) were used as secondary antibodies.

### Image analysis

Microscopic slides were imaged on a Zeiss AxioObserver.Z1 7 inverted fluorescent microscope equipped with an ApoTome to increase z-plane resolution of images. For OHSCs, X20 tiled images covering the entire hippocampal slices were taken for analysis of PFF-seeded aggregation in α-syn wt- vs. α-syn T64E/T72E- expressing slices. The same imaging settings were used for all images, and staining with aggregation-specific anti-α-syn, MJF-14–6–4–2, was used to define α-syn inclusions. Images were analyzed in FIJI (Fiji Is Just ImageJ, NIH), and the total aggregate area was normalized to the tissue area for each slice. First, DAPI staining was used to define the tissue area (50 pixels enlargement of the DAPI selection). Subsequently, trainable Weka segmentation was used to define aggregates, with a threshold of 0.50 in the created probability map (87). The Weka segmentor was trained on a selection of images with and without aggregates from slices expressing both α-syn wt- and α-syn T64E/T72E to ensure satisfactory segmentation ability. Aggregates were analyzed using FIJI's particle analyzer, with a size of 50 to 5000 pixel^2^. 4 to 6 slices were included in each group/time point.

### Statistics

All statistical analyses were performed using GraphPad Prism 7 (Graph Pad software). Comparison of groups was done by one-way ANOVA or two-way ANOVA followed by Tukey's multiple comparison test. When comparing just two groups, student *t* test with Welch's correction for differences in SD between groups was applied. A *P*-value  < 0.05 was considered significant.

## Funding

The study was supported by Lundbeck Foundation grants R223-2015-4222 for PHJ, R248-2016-2518 for Danish Research Institute of Translational Neuroscience-DANDRITE, Nordic-EMBL Partnership for Molecular Medicine, Aarhus University, Denmark. Aarhus University, Parkinsonforeningen and Bjarne Saxhofs Fond.

The 11A5 antibody was a kind gift from Imago Pharmaceuticals.

The MS work was supported by NIH grants (U01 NS091272 and R01 AG056711 to J.Z. and M.S.) and in part by the University of Washington's Proteomics Resource (UWPR95794).

## Supplementary Material

pgac259_Supplemental_File

## Data Availability

All data are included in the manuscript and/or Supplemantary Matreial.
