# Supplementary material for: Protein kinase R dependent phosphorylation of α-synuclein regulates its membrane binding and aggregation
Source: PNAS Nexus. 2022 Nov 16;1(5):pgac259. doi: 10.1093/pnasnexus/pgac259 (PMC9802061; doi:10.1093/pnasnexus/pgac259)
Supplement: pgac259_Supplemental_File [file pgac259_supplemental_file.pdf]

# Supplementary material

# PKR dependent phosphorylation of α-synuclein regulates its membrane binding and aggregation

Authors
Lasse Reimer*[a,b], Hjalte Gram[a,b], Nanna Møller Jensen[a,b], Cristine Betzer[a,b], Li Yang[c], Lorrain Jin[c], Min Shi[c], Driss Boudeffa[d] , Giuliana Fusco[e], Alfonso De Simone[f], Deniz Kirik[g], Hilal A Lashuel[d], Jing Zhang[c,h] and Poul Henning Jensen*[a,b]
[a]Danish Research Institute of Translational Neuroscience - DANDRITE, Aarhus University, Denmark.
[b]Department of Biomedicine, Aarhus University, Denmark.
[c]Department of Pathology, University of Washington School of Medicine, Seattle, USA
[d]Laboratory of Molecular and Chemical Biology of Neurodegeneration, School of Life Sciences Brain Mind Institute, Ecole Polytechnique Fédérale de Lausanne (EPFL), Station 19, 1015, Lausanne, Switzerland.
[e]Centre for Misfolding Diseases, Department of Chemistry, University of Cambridge, CB2 1EW UK
[f]Department of Pharmacy, University of Naples, Via Montesano, 49 80131, Naples IT
[g]Brain Repair and Imaging in Neural Systems, Department of Experimental Medical Science, Lund University, BMC D11, 22184 Lund, Sweden
[h]Department of Pathology, Zhejiang University School of Medicine and the First Affiliated Hospital, Hangzhou, China
*Corresponding author
Email: lasse.reimer@dandrite.au.dk

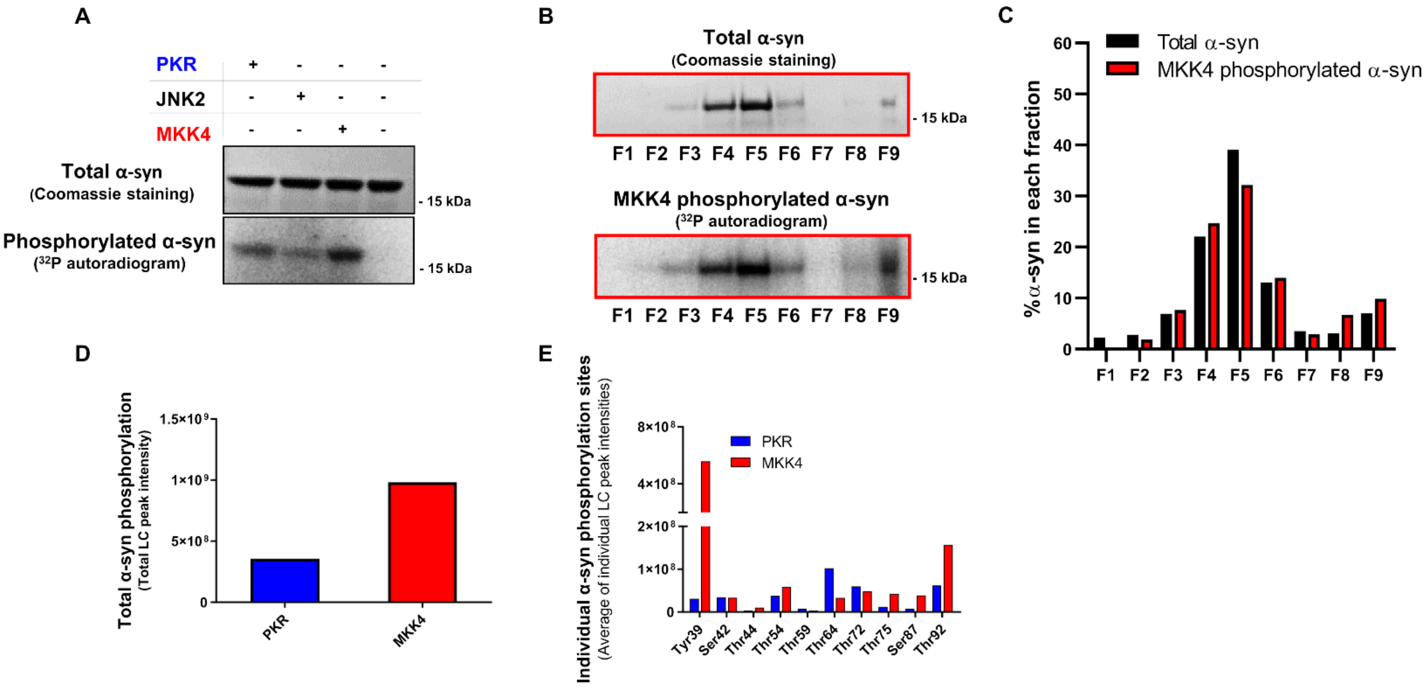


***Supplementary Figure 1. MKK4 phosphorylates α-syn S87A/S129A without affecting its liposome-binding. A)*** *Coomassie Blue gel staining and $^{32}P$ signal from PKR, JNK2 and MKK4 phosphorylated α-syn using autoradiography. α-syn S87A/S129A was incubated for 1 h at 30°C in phosphorylation buffer, with a 8:1 mixture of non-labeled and (γ-$P^{32}$)-labeled ATP (10 µM final concentration), either alone (lane 4) or together with recombinant human PKR (lane 1), recombinant human JNK2 (lane 2) or recombinant human MKK4 (lane 3).* ***B)*** *Coomassie Blue gel staining of total protein in fractions from flotation assay performed with 80:20 DMPG:DMPC liposomes and α-syn S87A/S129A in vitro phosphorylated by MKK4 (top panel). $^{32}P$ signal from phosphorylated α-synS87A/S129A using autoradiography of the gel in top panel (bottom panel).* ***C)*** *Quantification of total α-syn levels (black bars) or MKK4 phosphorylated α-syn (red bars) in each fraction of the flotation assay, based on Coomassie Blue stained gel staining and $^{32}P$ signal respectively. Y-axis demonstrates the percentage of the total α-syn or phosphorylated α-syn present in each fraction.* ***D)*** *Sum of signal from all α-syn phosphopeptides from α-syn wt in vitro phosphorylated by recombinant human PKR or MKK4. Protein samples were digested and phosphopeptides were enriched using titaniumdioxide (TiO2) affinity purification before mass spectrometry (MS) analysis. Label-free quantification was performed using peak areas of the identified peptides, and compared across different samples.* ***E)*** *Signal of individual α-syn phosphorylation sites from phosphopeptides of α-syn in vitro phosphorylated by recombinant human PKR or MKK4 using MS analysis and label-free quantification.*

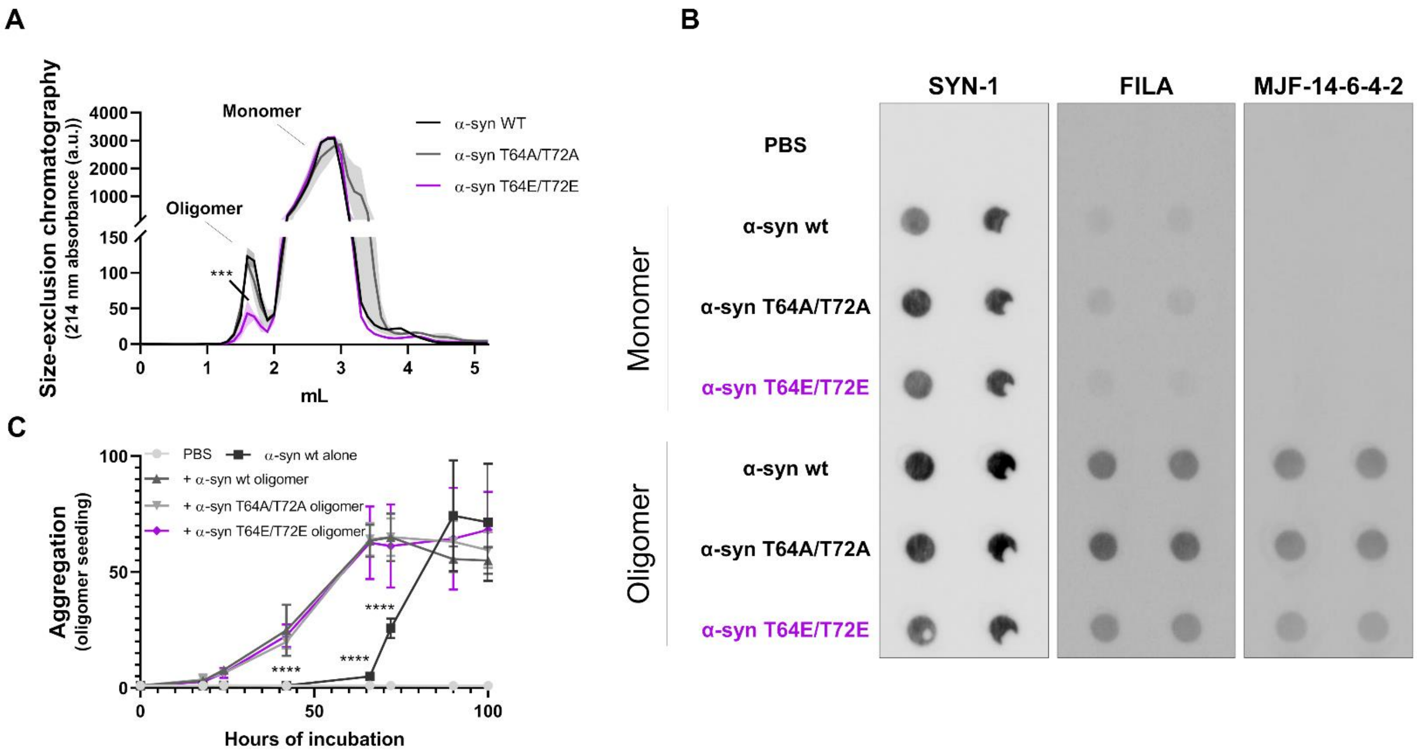


***Supplementary Figure 2. T64E/T72E phospho-mimicking α-syn oligomerizes less efficient, but its oligomers seed equally well aggregation of α-syn wt monomers. A)*** *α-syn oligomers formed by incubation of monomeric recombinant human at 12mg/mL under agitating conditions. α-syn oligomers were separated from monomers by size using size-exclusion chromatography (SEC). Yield of oligomers were calculated as area under curve based on three independent experiments for each of the three α-syn species (wt, T64A/T72A or T64E/T72E, n = 3, ***p < 0.001, based on one-way ANOVA followed by Tukey's multiple comparisons test).* ***B)*** *Representative image of antigenicity of PBS, or α-syn monomers or oligomers from the three α-syn species purified via SEC. These were tested with SYN-1 (total α-syn) or, FILA5 or MJF14 (aggregation specific) antibodies. Dots consist of 100ng protein spotted in duplicates (n = 3).* ***C)*** *Recombinant α-syn wt was incubated in PBS at a final concentration of 2 mg/mL at 37°C alone or in the presence of 0.2% oligomers from either of the three α-syn species (wt, T64A/T72A or T64E/T72E). ThT fluorescence signal was measured at 0h, 18h, 24h, 42h, 66h, 72h, 90h and 100h (excitation at 450 nm and emission at 486 nm) and normalized to ThT signal from 0h of incubation. Each sample was measured in duplicates and displayed as mean ± SD. Figure is representative of three independent experiments (n = 3, ****p < 0.0001, based on two-way ANOVA followed by Tukey's multiple comparisons test).*

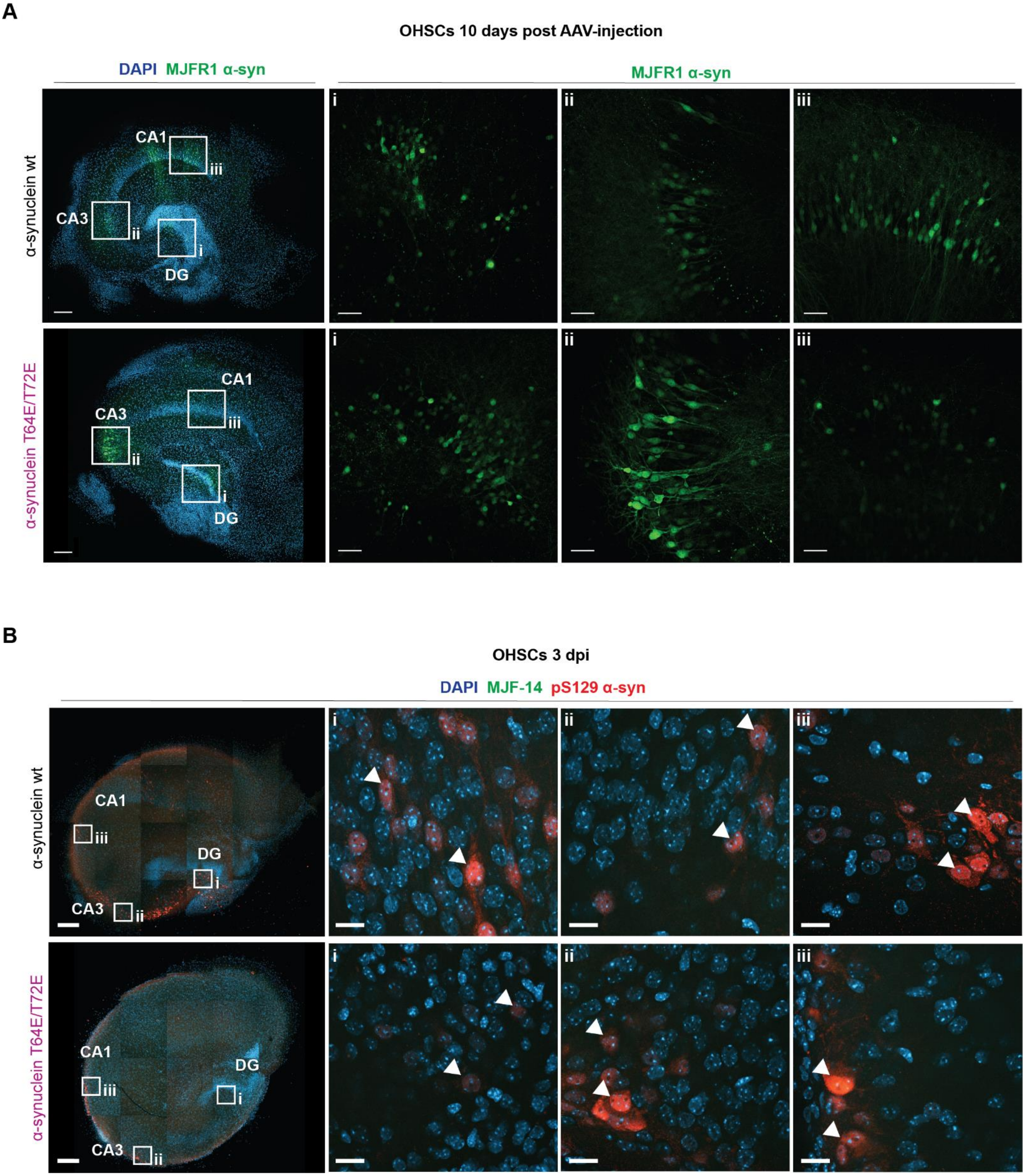


***Supplementary Fig. 3. AAV-mediated α-syn wt and α-syn T64E/T72E expression and phosphorylation in OHSCs***

***A)*** *Expression of α-syn wt (top panel) and α-syn T64E/T72E (bottom panel) in OHSCs at 10 days post virus injection, as detected by anti-human-α-syn MJFR1 (green) and DAPI (blue). Scale bars = 200 µm, insets = 50 µm. Representative images from 6 slices/group.* ***B)*** *Nuclear phospho-S129 expression in α-syn wt (top panel) and α-syn T64E/T72E (bottom panel) OHSCs at 3 days post PFF injection, before aggregation is evident. Cultures are stained for aggregated α-syn (MJF-14-6-4-2, green), phospho-S129 α-syn (11A5, red) and nuclei (DAPI, blue). Strong nuclear S129-phosphorylation is seen for both α-syn variants (examples indicated by arrowheads). Scale bars = 200 µm, insets = 50 µm.*
